# Determinants of G quadruplex-induced epigenetic instability in REV1-deficient cells

**DOI:** 10.15252/embj.201488398

**Published:** 2014-09-04

**Authors:** Davide Schiavone, Guillaume Guilbaud, Pierre Murat, Charikleia Papadopoulou, Peter Sarkies, Marie-Noëlle Prioleau, Shankar Balasubramanian, Julian E Sale

**Affiliations:** 1 Medical Research Council Laboratory of Molecular BiologyCambridge, UK; 2 Department of Chemistry, University of CambridgeCambridge, UK; 3 The Gurdon InstituteCambridge, UK; 4 Institut Jacques Monod, Université Paris Diderot-Paris 7Paris, France

**Keywords:** epigenetic memory, G quadruplex, histone modifications, replication, REV1

## Abstract

REV1-deficient chicken DT40 cells are compromised in replicating G quadruplex (G4)-forming DNA. This results in localised, stochastic loss of parental chromatin marks and changes in gene expression. We previously proposed that this epigenetic instability arises from G4-induced replication fork stalls disrupting the accurate propagation of chromatin structure through replication. Here, we test this model by showing that a single G4 motif is responsible for the epigenetic instability of the *BU-1* locus in REV1-deficient cells, despite its location 3.5 kb from the transcription start site (TSS). The effect of the G4 is dependent on it residing on the leading strand template, but is independent of its *in vitro* thermal stability. Moving the motif to more than 4 kb from the TSS stabilises expression of the gene. However, loss of histone modifications (H3K4me3 and H3K9/14ac) around the transcription start site correlates with the position of the G4 motif, expression being lost only when the promoter is affected. This supports the idea that processive replication is required to maintain the histone modification pattern and full transcription of this model locus.

## Introduction

The vertebrate genome is littered with sequences capable of forming secondary structures that can interfere with replication (Mirkin & Mirkin, 2007), of which the G quadruplex (G4) is one of the most intensively studied (Maizels & Gray, 2013). dG bases in G-rich sequences can form planar Hoogsteen base-paired rings (G quartets) that stack to form often highly thermodynamically stable secondary structures, G quadruplexes (Sundquist & Klug, 1989; Williamson *et al*, 1989). While it has been estimated that there are more than 300,000 sequences capable of forming G4s (G4 motifs) within the human genome (Huppert & Balasubramanian, 2005; Todd *et al*, 2005), the existence and relevance of these structures *in vivo* has been controversial. However, evidence is mounting from studies using specific antibody or small molecule-mediated detection methods to support their formation *in vivo* (Schaffitzel *et al*, 2001; Müller *et al*, 2010; Biffi *et al*, 2013; Lam *et al*, 2013; Henderson *et al*, 2014). Further, G4s have been implicated in the induction of both genetic and epigenetic instability particularly in cells lacking enzymes required for their unwinding and replication (Cheung *et al*, 2002; London *et al*, 2008; Ribeyre *et al*, 2009; Sarkies *et al*, 2010, 2012; Lopes *et al*, 2011; Piazza *et al*, 2012; Koole *et al*, 2014).

Whether some or all G4 motifs have a function is unknown. A subset of motifs have been linked to transcriptional regulation (Bochman *et al*, 2012) and to the specification of DNA replication origins (Besnard *et al*, 2012; Cayrou *et al*, 2012; Valton *et al*, 2014). However, it is also likely that many of these sequences do not play a direct role in genome regulation. Nonetheless, all potentially pose a considerable impediment to DNA replication, as they can block the progression of replicative polymerases (Woodford *et al*, 1994). It is therefore perhaps unsurprising that in the past few years multiple enzymes have emerged as important in G4 resolution and replication. In budding yeast, the major activity that counteracts G4 formation is the helicase PIF1 (Ribeyre *et al*, 2009; Paeschke *et al*, 2011, 2013). In higher eukaryotes, a broader range of enzymes is involved. These include the homologue of PIF1 (Sanders, 2010), other specialised helicases including FANCJ (Cheung *et al*, 2002; Kruisselbrink *et al*, 2008; London *et al*, 2008; Wu *et al*, 2008), WRN and BLM (Sun *et al*, 1998; Fry & Loeb, 1999; Mohaghegh *et al*, 2001) and the specialised DNA polymerases REV1 (Sarkies *et al*, 2010), Pol κ and Pol η (Bétous *et al*, 2009).

We have previously reported that cells lacking key G4 processing enzymes, including REV1, FANCJ, WRN and BLM, exhibit localised epigenetic instability in the vicinity of G4 motifs (Sarkies *et al*, 2010, 2012). We proposed a model to explain these observations in which failure to replicate G4 motifs in a timely manner leads to uncoupling of the replicative DNA helicase and DNA polymerase resulting in the formation of post-replicative gaps. The ultimate replication of these gaps and G4 motifs would then take place remote from the replication fork and thus from the supply of parental histones. As a consequence, only new, naïve histones that do not bear the post-translational modifications characteristic of the parental chromatin would be incorporated. Thus, we suggested, delayed chromatinisation of the gap would result in loss of the epigenetic marks carried by the histones resulting in changes in gene expression. Consistent with this model, we have observed loss of histone marks associated with transcriptionally repressed chromatin (e.g. H3K9me2) and with transcriptionally active chromatin (e.g. H3K4me3 and H3K19/14ac) in loci containing G4 motifs (Sarkies *et al*, 2010, 2012).

Here, we explore some predictions of this model by expanding our study of an actively expressed locus, *BU-1*, in which epigenetic instability can be monitored directly by cytometry (Sarkies *et al*, 2012). We show that the unstable expression of this locus in REV1-deficient cells is explained by a single G4 motif located 3.5 kb downstream of the transcription start site (TSS). We manipulate *BU-1* to address a series of questions: (i) Is epigenetic instability induced by G4 motifs on both the leading and lagging strand? (ii) How do the *in vitro* biophysical characteristics of G4 motifs relate to their ability to cause epigenetic instability in REV1-deficient cells? and (iii) How does the distance of the G4 motif from the TSS influence the histone modification pattern and stability of expression of the *BU-1* locus? Together, the answers help build a picture of the interplay between the replication fork, the G4 motif and the epigenetic landscape of the locus. Our data also provide direct evidence of a link between loss of histone modifications due to interruption of DNA replication and inability to maintain high-level gene expression.

## Results

### Organisation and replication of the *BU-1* locus in DT40

**Figure 1:**
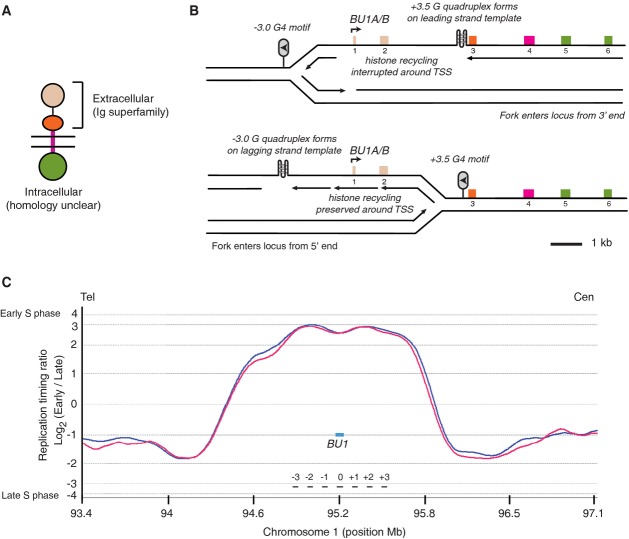
Replication of the *BU-1* locus Cartoon of the domain structure of the Bu-1 protein, adapted from UniProt (http://www.uniprot.org) protein ID Q90746 (Tregaskes *et al*, 1996).The predicted impact of the −3 and +3.5 G4 motifs on replication of the *BU-1* locus (ENSGALG00000015461). The exons are coloured to reflect their contribution to the domain structure of the protein as shown in (A). In the upper panel, the +3.5 G4 motif has formed a G quadruplex structure, which has stalled the leading strand polymerase resulting in the formation of a post-replicative gap. This results in a zone in which histone recycling is interrupted and therefore in which the histone modifications characteristic of the parental chromatin are lost, in this case H3K4me3 and H3K9/14ac. In the lower panel, a fork approaching from the 5′ end encounters a G quadruplex structure formed by the −3 G4 motif on the lagging strand template. This does not result in formation of a long post-replicative gap or in any perturbation to histone recycling and mark propagation around the TSS of *BU-1*.The *BU-1* locus is at the centre of an early replicating domain. S-phase cells were BrdU pulse-labelled, sorted into two fractions (Early-S and Late-S). BrdU-DNA from early and late fractions was differentially labelled and cohybridised to a chicken whole-genome microarray at a density of one probe every 5.6 kb. The log_2_ ratios (Early/Late) of the abundance were smoothed and are shown for two independent experiments. The timing analysis reveals that the *BU-1* locus replicates early in S phase but lies between two regions replicated just a little earlier. The markers −3 to +3 indicate the qPCR primers used to validate the array data (Supplementary Fig S1 and Supplementary Table S1).

Bu-1 is an avian homodimeric glycoprotein encoded by the *BU-1* (also known as *B6.1*) locus (Fig 1A). Bu-1 is found on bursal B cells and may play a role in regulating cell survival during development (Tregaskes *et al*, 1996; Funk *et al*, 1997). Two allelic variants of the *BU-1* locus have been defined, producing antigenically distinguishable proteins, Bu-1a and Bu-1b (Funk *et al*, 1997). The chicken DT40 cell line is derived from an F_1_ hybrid Hyline SC strain and is heterozygous for the *BU-1A* and *BU-1B* alleles. We have previously shown that expression of Bu-1a is unstable in DT40 mutants lacking enzymes involved in replicating G4s, including the specialised DNA polymerase REV1 and DNA helicases FANCJ, WRN and BLM (Sarkies *et al*, 2012). This instability is epigenetic rather than genetic in origin as the changes in gene expression and associated changes in histone post-translational modifications are not accompanied by any changes in the DNA sequence (Sarkies *et al*, 2012). The *BU-1* locus contains two clearly identifiable G4 motifs (Sarkies *et al*, 2012). One is 3.5 kb downstream of the TSS between exons 2 and 3 (hereafter referred to as the +3.5 G4 motif), with its G-rich sequence on the coding strand. The other is 3 kb upstream of the TSS (the −3 G4 motif) and in the same orientation (Fig 1B). We previously speculated that instability of Bu-1a expression in REV1-deficient cells would be dependent on the +3.5 G4 motif (Sarkies *et al*, 2012), since this structure would form on the leading strand template of a replication fork heading towards the TSS from the 3′ end of the locus (Fig 1B). In contrast, the G-rich strand of the −3 G4 would form on the lagging strand template of a fork heading towards the TSS from the 5′ end of the locus (Fig 1B). Exposure of single-stranded DNA following fork arrest by a replication impediment on the lagging strand should be limited by Okazaki priming. Since the length of Okazaki fragments approximates to a single nucleosome (Smith & Whitehouse, 2012), lagging strand arrest should not result in epigenetic instability.

Although it has been suggested that G4 motifs contribute to the specification of replication origins (Besnard *et al*, 2012; Cayrou *et al*, 2012; Valton *et al*, 2014), there are no significant replication origins within the *BU-1* locus (Supplementary Fig S1A). The locus lies within a short (c. 50 kb) early constant timing region and is surrounded by two stretches replicated just before it (Fig 1C and Supplementary Fig S1B) (Sarkies *et al*, 2010). This indicates that the *BU-1* locus is either passively replicated equally from the left or from the right, or that is replicated by multiple, synchronous internal initiations (Guilbaud *et al*, 2011). Given that the average inter-origin distance in DT40, determined by molecular combing, is 76 +/− 7 kb (Supplementary Fig S1C), it is most likely that the locus is bidirectionally replicated, with the fork entering from the 3′ end of the locus in about 50% of S phases.

### Epigenetic instability of Bu-1a in *rev1* cells is dependent on the +3.5 G4 motif

**Figure 2:**
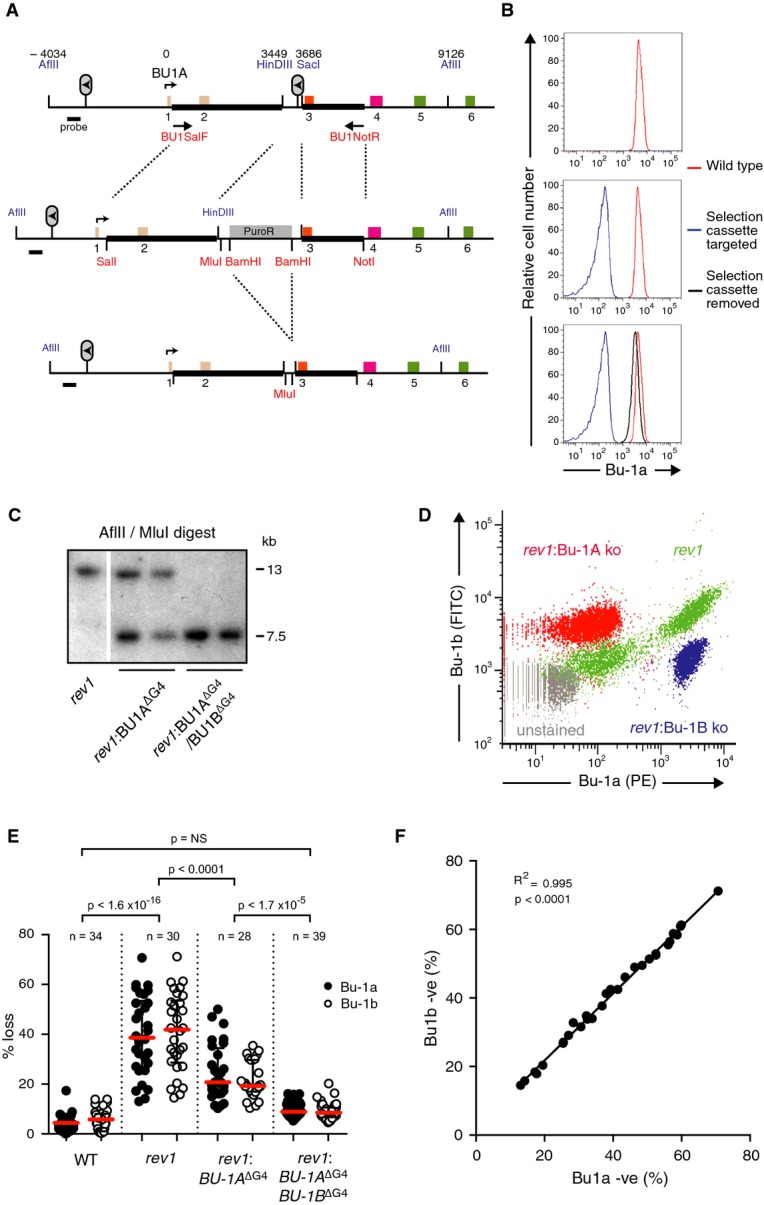
Epigenetic instability of Bu-1a is dependent on the +3.5 G4 motif Gene targeting strategy used to delete or replace the +3.5 G4 motif of the *BU-1* locus. The six exons are colour-coded according to the domains of the protein shown in Fig 1A. BU1SalF and BU1NotR are the primers used to amplify the genomic region used in the construct (thick black bars). Restriction sites introduced into the construct are shown in red, and endogenous sites of importance are shown in blue. Numbers above the map indicate the position of key features, relative to the transcriptional start site.Bu-1a expression at the three stages of targeting shown in (A).Confirmation of targeting of the *BU-1A* and *BU-1B* alleles by Southern blotting. Sizes of genomic fragments for the endogenous locus in the *rev1* background and the disrupted locus are shown.Correlation of loss of Bu-1a and Bu-1b expression in *rev1* cells (green) compared with the effect of genetic abrogation of expression of Bu-1a (red) and Bu-1b (blue). Unstained cells in grey.Fluctuation analysis showing loss of Bu-1a and Bu-1b expression after deletion of the +3.5 G4 motif on the *BU-1A* allele and after its deletion from both *BU-1A* and *BU-1B*. Red bar = median loss; whiskers = interquartile range; *P*-values for compared Bu-1a loss distributions calculated with Fisher's exact test with a bin size of 20%.Correlation of the percentage of Bu-1a and Bu-1b loss variants in individual *rev1* clones.

We first tested whether the +3.5 G4 motif is indeed responsible for the instability of Bu-1a expression. We created a targeting construct designed to replace the G4 motif with a selection cassette flanked by loxP recombination sites (Fig 2A). When integrated, this construct inactivated surface Bu-1a expression, due to insertion of the selection cassette into the middle of the gene (Fig 2B). However, upon treatment with Cre recombinase, expression of Bu-1a was restored to wild-type levels (Fig 2B and Supplementary Fig S2). We confirmed targeting of the locus by FACS (Fig 2B) and Southern blotting (Fig 2C).

To assess the effect of deletion of the +3.5 G4 motif in the *BU-1A* locus of *rev1* cells, we performed a fluctuation analysis for Bu-1a loss (Supplementary Fig S3), by expanding individual Bu-1a^high^ *rev1*:*BU-1A*^*ΔG4*^ cells for 20 generations and then measuring the proportion of each clone that had become Bu-1a^low^ by flow cytometry. We have previously shown that loss of Bu-1a protein expression is explained by loss of Bu-1a transcript and that there is a direct correlation between Bu-1a transcript and the percentage of Bu-1a loss variants in a population (Sarkies *et al*, 2012). We compared the frequency of Bu-1a^low^ variants with *rev1* cells harbouring an intact *BU-1A* +3.5 G4 motif. Interestingly, the rate at which Bu-1a^low^ variants were generated by the *rev1*:*BU-1A*^*ΔG4*^ clones was reduced relative to the parental *rev1* mutant, but was still well above the background level observed in wild-type clones (Fig 2E). Since the G4 motif on *BU-1B* was still intact, one possible explanation was interallelic crosstalk. Supporting this idea, the percentage of Bu-1a^low^ and Bu-1b^low^ variants in individual clones was closely correlated (Fig 2F) as was the loss of Bu-1a and Bu-1b expression at the level of single cells (Fig 2D, green plot). This effect was not due to a requirement to form Bu1a-b heterodimers because complete loss of expression of Bu-1a due to the introduction of a selection cassette has no effect on Bu-1b expression, and vice versa (Fig 2D). To test the possibility of a *trans* effect between the two alleles, we additionally removed the +3.5 G4 motif on *BU-1B* and performed a fluctuation analysis for generation of Bu-1a^low^/b^low^ cells. Removing the +3.5 G4 motif on both alleles resulted in Bu-1a expression becoming stable (Fig 2E). The basis for this interallelic crosstalk remains unclear, although it is reminiscent of the linked expression of alleles described in plants and flies (Hollick, 2012; Kassis, 2012). We are undertaking further experiments to understand the mechanism underlying this phenomenon. However, the remaining experiments presented here are carried out using sequences of interest knocked into the *BU-1A* allele of cells with the genotype *rev1: BU-1A*^*ΔG4*^*/BU-1B*^*ΔG4*^, thus removing any interallelic effects.

### Instability of Bu-1a expression requires formation of the +3.5 G4 motif on the leading strand template

**Figure 3:**
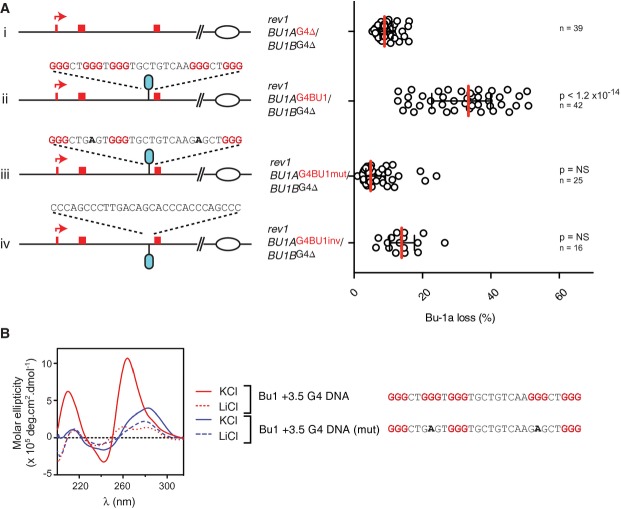
Epigenetic instability of Bu-1a is dependent on the +3.5 G4 motif The cartoons show the manipulation of the *BU-1A* locus in which the +3.5 G4 motif has already been deleted from both the *BU-1A* and *BU-1B* allele. Only the first three exons are shown along with the sequence reintroduced in the position of the original +3.5 G4 motif. (i) *BU1A* with the +3.5 kb G4 DNA deleted. (ii) *BU1A* with the +3.5 kb G4 DNA reintroduced. (iii) *BU1A* with the +3.5 kb G4 DNA reintroduced with two point mutations that abolish intramolecular G quadruplex formation *in vitro* (Fig 3B). (iv) *BU1A* with the +3.5 kb G4 DNA reintroduced inverted such that it would be present on the lagging strand template for a replication fork entering from the right of the locus. To the right is the fluctuation analysis of Bu-1a loss for each mutant. Each point represents the percentage of Bu-1a^low^ variants in clones expanded for 20 generations. Red bar = median loss; whiskers = interquartile range; *P*-values that each distribution is different from control (i) calculated with Fisher's exact test with a bin size of 20%. NS = not significant (*P* < 0.001).Circular dichroism spectroscopy of the native *BU-1* +3.5 G4 motif (red line) and a version mutated to prevent intramolecular G4 formation (blue line) carried out in KCl (solid line) and LiCl (dashed line).

Having demonstrated that removal of the +3.5 G4 motif leads to stable Bu-1 expression, we next went on to test the properties of the G4 motif required to trigger epigenetic instability. Reintroduction of the wild-type sequence into the *BU-1A* allele resulted in renewed stochastic loss of Bu-1a expression (Fig 3A), confirming that the presence of the wild-type G4 motif is sufficient to induce instability of expression. However, introduction of the G4 motif with two point mutations that render it incapable of forming an intramolecular G4 structure *in vitro* (Fig 3B) did not induce instability of Bu-1a expression (Fig 3A). The stabilisation of Bu-1a expression following disruption of the +3.5 G4 motif is consistent with our previous hypothesis that epigenetic instability of the locus results from the formation of a secondary structure that stalls the leading strand of a fork entering the locus from the right (Fig 1B). To further test this, we reintroduced the +3.5 G4 motif in an inverted orientation such that the G-rich strand would form on the lagging strand template of a fork entering the locus from the right. The presence of the inverted G4 motif did not result in significant instability of expression (Fig 3A).

### Epigenetic instability of BU-1 in *rev1* cells does not correlate with *in vitro* G4 thermal stability

**Figure 4:**
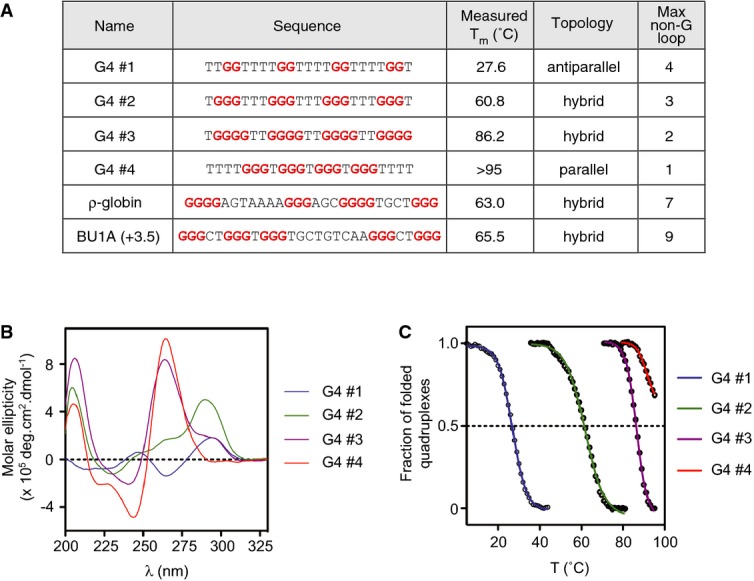
Biophysical characterisation of the library of G4 motifs Summary data table. G4#1–4 are model G4 motifs designed to have a range of melting temperatures. The *in vitro* topology was determined by circular dichroism in the presence of K^+^, shown in (B), and melting temperature is derived from the UV melting curves shown in (C).Circular dichroism spectra for G4#1–4 in K^+^. CD spectra for the ρ-globin and Bu-1a G4 DNAs have been determined previously (Barski *et al*, 2007; Sarkies *et al*, 2010, 2012).UV melting curves of G4#1–4. The T_m_ for the ρ-globin and Bu-1a G4 DNAs have been determined previously (Sarkies *et al*, 2010, 2012).

We next asked whether the ability to induce epigenetic instability of *BU-1* in *rev1* cells is peculiar to the naturally occurring +3.5 G4 motif, or whether other G4 motifs could produce the same effect. To that end, we designed a set of four G4 motifs that form structures of increasing *in vitro* thermal stability (Fig 4A), predicting that thermodynamically more stable structures would lead to greater effects. Under conditions favouring intramolecular G4 formation, oligodeoxynucleotides of each of these sequences formed a G4 *in vitro* in KCl as assessed by circular dichroism spectroscopy (Fig 4B). G4#1 adopts a predominantly antiparallel conformation and G4#4 predominantly parallel. G4#2 and G4#3 exhibit spectra consistent with a hybrid mixture of conformations (Fig 4A and B). UV melting curves revealed a range of T_m_s of between 27.6°C for G4#1, a sequence predicted to form only a two-stack G4, to > 95°C for G4#4 (Fig 4A and C). Each of these sequences was introduced into the *BU-1A* allele of *rev1: BU-1A*^*ΔG4*^*/BU-1B*^*ΔG4*^ cells as a single copy in the same position and orientation as the natural +3.5 G4 motif, and fluctuation analysis for the generation of Bu-1a^low^ variants performed. In addition, we compared the effect of the G4 motif from the ρ-globin locus (Sarkies *et al*, 2010) and the native +3.5 Bu1a G4 motif, both of which have similar *in vitro* melting temperatures to G4#2, but are more complex, with five G runs and longer non-G loops.

**Figure 5:**
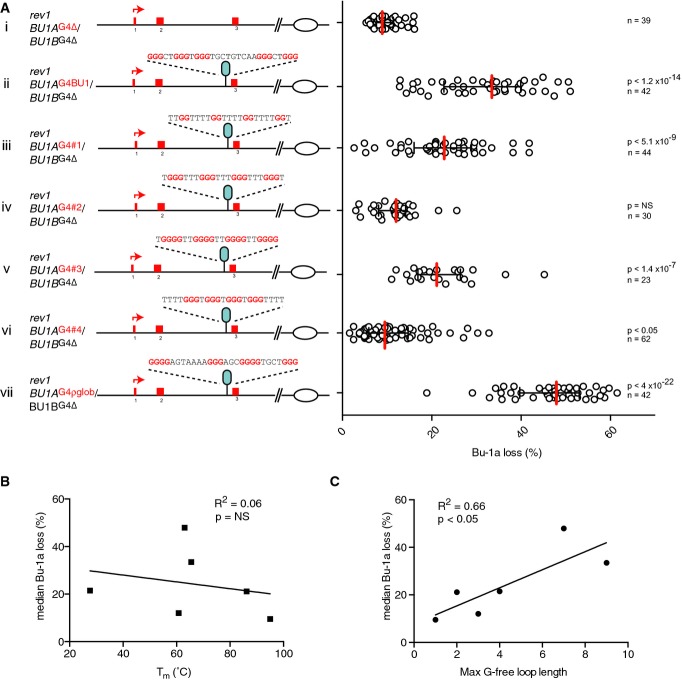
Dependency of Bu-1a loss on G4 motifs of differing sequence in the +3.5 position Bu-1a loss in mutants harbouring different G4 motifs at +3.5 kb from the TSS. (i) *rev1* cells with the G4 DNAs removed from both the *BU-1A* and *BU-1B* alleles. (ii) Knock-in of the wild-type +3.5 Bu-1 G4 motif. (iii–vi) Knock-ins of G4#1–4. (vii) Knock-in of the ρ-globin intron 2 G4 motif (Sarkies *et al*, 2010). Red bar = median loss; whiskers = interquartile range; *P*-values that each distribution is different from control (i) calculated with Fisher's exact test with a bin size of 20%. NS = not significant (*P* < 0.001).Pearson correlation of the median level of Bu-1a loss with the *in vitro* melting temperature of oligonucleotides of each G4 motif.Pearson correlation of the median level of Bu-1a loss with the maximum non-G loop length of each G4 DNA sequence under test.

Surprisingly, G4#1 induced significant Bu-1a instability (Fig 5A), despite having a predicted stack height of just two G quartets. Further, the sequence forming the most stable G4 structure *in vitro*, G4#4, resulted in relatively few Bu-1a^low^ variants (Fig 5A and Supplementary Fig S2), while G4#2 and the ρ-globin G4 motif induced very different levels of instability, despite both having very closely matched melting temperatures. Overall, we did not observe any significant correlation between Bu-1a loss and T_m_ over the six lines examined (Fig 5B). Rather, there is a general relationship between the maximum non-dG loop length in the quadruplex-forming sequence and Bu-1a loss (Fig 5C). We return to these relationships in the Discussion.

### A leading strand G4 motif can induce instability of expression from up to 4 kb from the TSS

**Figure 6:**
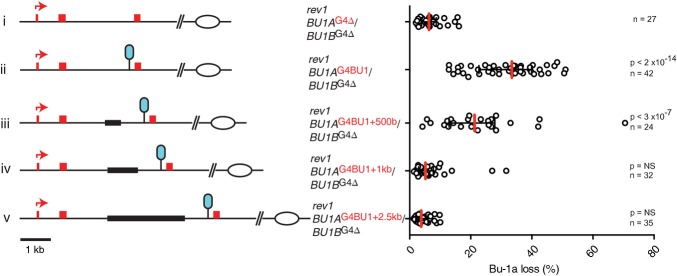
Dependence of loss of Bu-1a expression and histone marks around the TSS on the distance between the +3.5 G4 motif and the TSS Bu-1a loss is reduced as the G4 motif is moved further from the TSS. The cartoons show the position of the +3.5 G4 motif following manipulation of the *BU-1A* locus in which the natural +3.5 G4 motif has already been deleted from both the *BU1A* and *BU1B* allele (i). Only the first three exons are shown. (ii) The +3.5 G4 motif reintroduced into its native position. (iii–v) The +3.5 G4 DNA reintroduced with additional spacers placing it 4 kb, 4.5 kb and 6 kb from the TSS. To the right of the cartoons are the corresponding fluctuation analyses for Bu-1a loss. Each point represents the percentage of Bu-1a^low^ variants from single Bu-1a^high^ cells expanded for 20 generations. Red bar = median loss; whiskers = interquartile range; *P*-values that each distribution is different from control (i) calculated with Fisher's exact test with a bin size of 20%. NS = not significant (*P* < 0.001).

Our original model invoked the formation of post-replicative gaps that extend several kilobases beyond a stalled replication fork towards and over the TSS (Fig 1B, Sarkies *et al*, 2010). We therefore hypothesised that moving the G4 motif further away from the TSS should decrease the probability that the gap would be long enough to reach the TSS, resulting in decreased generation of Bu-1a^low^ variants. To test this idea, we knocked in spacers, comprising sequence already present in the second intron of the *BU-1* locus, to position the +3.5 G4 motif a further 500, 1,000 and 2,500 bp away from the TSS. We then examined the formation of Bu-1a^low^ variants in a fluctuation analysis (Fig 6A). Compared with cells in which the +3.5 G4 motif had been reintroduced in its native position, reintroduction of the G4 motif just 500 bp further away from the TSS (4,000 bp from the TSS) reduced the rate at which Bu-1a^low^ variants were generated by about 50% (Fig 6A). Bu-1a loss diminished further, to background levels, when the G4 motif was placed 4,500 bp or 6,000 bp away (Fig 6). These observations suggest a maximum range of influence of the G4 motif of about 4.5 kb.

### The position of the G4 motif determines the pattern of H3K4me3 and H3K9/14ac independently of gene expression

**Figure 7:**
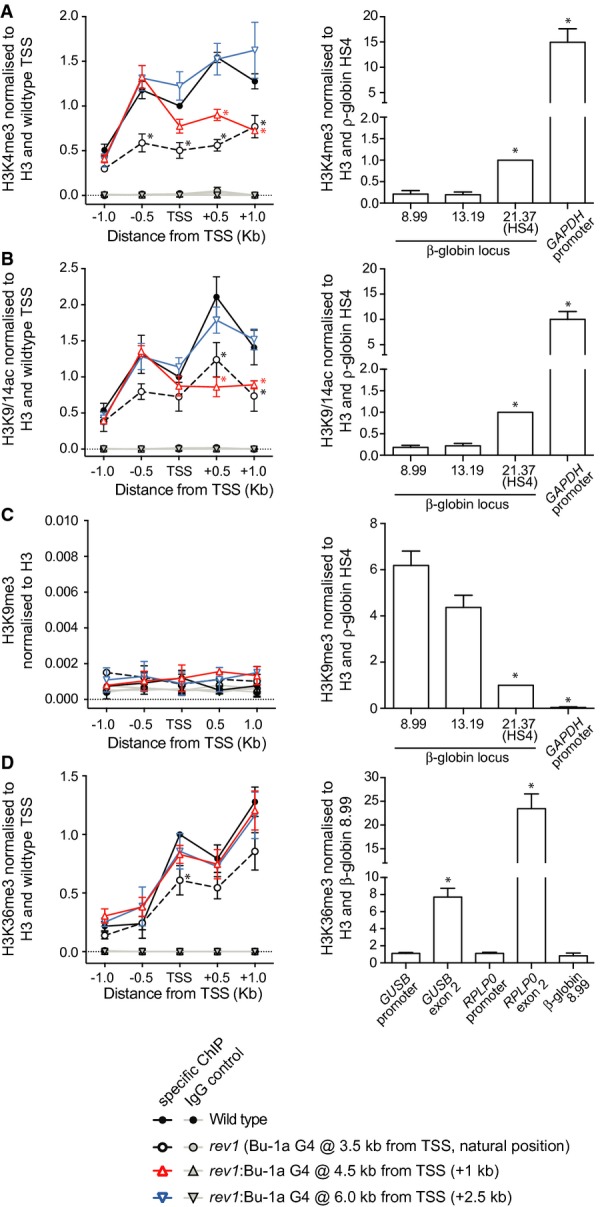
Alterations in the chromatin landscape around the *BU-1* TSS as a function of the position of the +3.5 kb G4 motif A–D H3K4me3 (A), H3K9/14ac (B), H3K9me3 (C) and H3K36me3 (D) around the TSS of *BU-1* as a function of the distance from the TSS to the +3.5 G4. Right panels: specific ChIP signal. Solid black line: wild type; dashed black line: *rev1* cells with the Bu-1a +3.5 G4 DNA knocked out on both alleles and then knocked back into its original position on the *BU-1A* allele; red line: G4 motif knocked back in at +4.5 kb from TSS; blue line: G4 motif knocked back in at +6 kb from TSS. The specific signal was normalised to total H3 and then to the signal at the TSS in wild-type cells, with the exception of H3K9me3 where there was no significant enrichment above IgG control indicated by the grey lines and symbols. H3 density is shown in Supplementary Fig S4. Left panels: ChIP antibody controls. 8.99 and 13.19 are primer pairs located within the constitutive heterochromatin between the ρ-globin and folate receptor genes at the 5′ end of the β-globin locus, and HS4 is an insulator element separating this heterochromatin from the ρ-globin gene and is enriched in ‘active’ histone marks (Sarkies *et al*, 2010). GAPDH is a fully active locus. For H3K36me3, the controls are two active loci in which enrichment at the promoter is compared with the body of the gene. Results are normalised to the signal from the ρ-globin 8.99 primer pair. Data are presented as an average of three independent immunoprecipitations from separate pools of cells of the indicated genotype with enrichment monitored by qPCR in triplicate for each immunoprecipitation (i.e. each point consists of nine technical replicates). Error bars = SD of the three biological replicates. For the *BU-1* ChIP, data points that are significantly different (*P* < 0.01, Mann–Whitney test) from wild type are indicated with an asterisk. For the controls, significance was tested against the β-globin 8.99 primer pair.

We next examined the influence of the position of the G4 motif in the *BU-1A* locus had on the pattern of histone modification around the TSS. In wild-type cells, H3K4me3 and H3K9/14ac are enriched around the TSS in the biphasic pattern characteristic of actively transcribed loci (Barski *et al*, 2007) (Fig 7A and B). REV1-deficient cells show significantly reduced enrichment of these modifications at the *BU-1A* promoter but no increase in the H3K9me3 (Fig 7C), a mark characteristic of heterochromatinised loci, consistent with our previous observations (Sarkies *et al*, 2012). Further, loss of histone modifications around the *BU-1A* TSS in *rev1* cells and reduced expression is not associated with any change in H3 density (Supplementary Fig S4) or chromatin accessibility (Supplementary Fig S5) also supporting there being no increase in chromatin compaction. Moving the G4 motif 1 kb further away from the TSS, to +4.5 kb, results in loss of H3K4me3 and H3K9/14ac in the body of the gene (+0.5 to +1.0 kb) but, surprisingly, not 5′ of the TSS (−0.5 kb) (Fig 7A and B), despite there being no reduction in Bu-1a expression or increased generation of Bu-1a^low^ variants (Fig 6). Moving the G4 motif to +6 kb from the TSS restored the wild-type pattern of both modifications (Fig 7A and B) and stable expression of the locus (Fig 6). In neither case was there any significant change in overall H3 density or in mRNA levels relative to wild type (Supplementary Fig S4). H3K36me3 modification is enriched in the body of expressed genes (Bannister *et al*, 2005; Barski *et al*, 2007). Its incorporation is coupled to transcription elongation (reviewed in Henikoff & Shilatifard, 2011), and it has recently been shown in yeast to limit histone turnover during transcription (Venkatesh *et al*, 2012). Interestingly, H3K36me3 was only reduced when the G4 was in its natural +3.5 kb position and loss of Bu-1a expression is high (Fig 7D) consistent with the maintenance of this mark being coupled to transcriptional activity rather than the position of the G4 motif. Together, these data suggest that, in this locus, maintenance of H3K4me3 requires processive replication and that the retention of this modification at the promoter is required for wild-type levels of expression.

## Discussion

In this study, we have exploited a genetically tractable system to explore the relationship between G4 motifs and replication-dependent instability of gene expression. In doing so, we have simultaneously gained insight into the role of processive replication in maintaining transcriptional states and created a sensitive assay that monitors problems with the replication of G4-forming DNA sequences *in vivo*.

The stochastic loss of expression of the actively transcribed *BU-1* locus in REV1-deficient cells is dependent on the presence of a single G4 motif located at a considerable distance (3.5 kb) from the transcriptional start site. Although some G4 motifs have been linked with the regulation of gene expression (reviewed in Maizels & Gray, 2013), we consider it unlikely that this G4 motif forms part of an intragenic regulatory element. Bu-1a expression in wild-type cells is the same and is stable, whether or not the +3.5 G4 motif is present (Supplementary Fig S6), suggesting that it has no direct influence on transcription under normal circumstances. Rather, several lines of evidence suggest that the instability of Bu-1a expression is replication dependent: (i) the stochastic nature of the switching to a low expression state, (ii) the dependence on the orientation of the G4 motif and (iii) the genetic dependence on enzymes, such as REV1 and FANCJ, that promote processive replication through G4 motifs and other impediments. Further, our simulation of Bu-1a loss as a replication-dependent phenomenon closely matches our experimental observations (Supplementary Fig S7).

### REV1 and the replication of G quadruplex-forming DNA

While there have been significant advances in the detection of G4 structures *in vivo* in recent years (Müller *et al*, 2010; Biffi *et al*, 2013; Lam *et al*, 2013; Henderson *et al*, 2014), these approaches are not yet sensitive enough to reliably detect the likely transient formation of a single G4 of the type under study here. The instability of the Bu-1a locus provides a sensitive readout for delayed G4 replication and will facilitate the study of the replication of these sequences *in vivo*.

Loss of REV1 does not perturb overall fork rates in the absence of damage, lead to formation of excessive single-stranded DNA or cause spontaneous checkpoint activation (Edmunds *et al*, 2008; Jansen *et al*, 2009). It is thus unlikely that there is greater opportunity for G4 formation ahead of the replication fork than in wild-type cells. Rather, we propose that the epigenetic instability arises because of delayed replication of structures that would normally be promptly resolved. The mechanism by which REV1 facilitates the replication of G4s is still not completely understood. We have previously implicated the Y-family polymerase-binding C-terminus of the protein but also suggested that the catalytic activity may play a role as well (Sarkies *et al*, 2010). An interesting recent study has demonstrated that the catalytic core of REV1, which has a marked capacity to bind single-stranded DNA (Masuda & Kamiya, 2006), is able to disrupt G quadruplex DNA directly (Eddy *et al*, 2014). This suggests that REV1 may contribute to melting G quadruplexes by a non-enzymatic mechanism perhaps similar to RPA (Qureshi *et al*, 2012). In turn, this may explain why cells particularly need REV1 for prompt replication of G4s with longer potential single-stranded loops. A surprising result is that a G4 motif, G4#1, with an *in vitro* thermal stability below the culture temperature of the cells can nonetheless give rise to significant instability of Bu-1a expression. This could argue against the formation of a ‘structure’ *in vivo* being responsible for the Bu-1a expression instability. However, it may also reflect a complex relationship between the *in vitro* melting temperature of an oligonucleotide in artificial conditions and *in vivo* structure-forming behaviour of the sequence in the context of a replication fork, a topic that requires further exploration.

We have suggested that a key consequence of the delayed replication of the +3.5 G4 motif in *rev1* cells is the uncoupling of the helicase and leading strand polymerase to create of post-replicative gap. Our Monte Carlo simulation (Supplementary Fig S7) suggests that the frequency of events that lead to loss of Bu-1a expression is low, in the order of 0.02, and, despite employing a variety of approaches, we have not been able, thus far, to detect directly an enrichment of single-stranded DNA in the *BU-1* locus of *rev1* cells. It is possible that it has a relatively short half-life. The fact that Bu-1a is likely to be replicated bidirectionally would mean that a single-stranded region formed by a fork from the 3′ end of the locus arresting at the +3.5 G4 may be rapidly replicated by a fork arriving from the 5′ end of the locus, but with the parental nucleosomes already displaced, thereby ‘fixing’ the mark loss.

Our data confirm the prediction of our model that epigenetic instability is dependent on interrupting leading strand replication. While we have previously argued that REV1 may act preferentially on the leading strand (Sale *et al*, 2009), it is also possible in this context that the leading strand bias reflects Okazaki fragment repriming, limiting the extent of post-replicative gaps on the lagging strand. It will therefore be interesting to test this hypothesis in other mutants such as FANCJ, which has previously been proposed to unwind G4s preferentially on the lagging strand (Cheung *et al*, 2002; Schwab *et al*, 2013).

### Processive DNA replication as a requirement for epigenetic maintenance of transcriptional memory

How could maintaining a pre-existing transcriptional state depend on processive replication? Fork arrest at tight protein–DNA complexes in *S. pombe* can lead to Sir-dependent heterochromatinisation and silencing (Dubarry *et al*, 2011). However, it is unlikely that a similar mechanism is operating in *BU-1* as we do not observe any increase in H3K9me3 (Fig 7C) or increased compaction of the locus in *rev1* cells (Supplementary Fig S5), suggesting that loss of Bu-1a expression is not a consequence of active heterochromatinisation.

Stable expression of the *BU-1* locus is sensitive to the position of the G4 motif relative to the TSS, consistent with a fixed zone in which histone marks are disrupted and that is defined by the position of the G4 motif (Supplementary Fig S8). Thus, when the G4 motif is placed +4.5 kb from the TSS, we observe loss of H3K4me3 and H3K9/14ac within the body of the gene, despite expression indistinguishable from wild-type cells. This result is consistent with the pattern of histone modifications being directly influenced by the position of the replication stall rather than by transcription. It also suggests that the presence of a ‘wild-type’ level of H3K4me3 and H3K9/14ac within the body of the gene is not essential for its full and stable expression. Interestingly, H3K36me3 is diminished only when the G4 motif is in the +3.5 kb position and H3K4me3 is lost at the promoter and expression of Bu-1a is unstable, suggesting that H3K36me3 is indeed likely to correlate with transcriptional activity. Together, these data suggest that H3K4me3 at the promoter of *BU-1* may be required to maintain fully active gene expression. Experiments in *Dictyostelium* and *Xenopus* have also provided evidence that H3K4me3 is required to maintain fully active transcription through cell division (Ng & Gurdon, 2008; Muramoto *et al*, 2010). Importantly, H3K4me3 is also well placed to be a primary epigenetic mark as it can promote the installation of other modifications conducive to transcription such as H3K9 acetylation (Pray-Grant *et al*, 2005).

However, the mechanism by which H3K4me3 might be maintained through replication remains unclear. Here, we show that maintenance of this mark is sensitive to loss of processive replication. This could be due to disruption of an active process for its installation linked to the replisome, or disruption of the recycling of the mark from the parental to daughter strands. Recombination induced by collision of replication forks with undisplaced RNA polymerase II has been proposed to interrupt the replication-dependent heterochromatinisation of pericentromeric repeats by the fork-associated Rik1 complex (Zaratiegui *et al*, 2011). However, we are not aware of any analogous mechanism of fork-associated installation of marks associated with active transcription, and such a model would not readily explain the close relationship between the position of the G4 motif and the loss of marks at the promoter. Instead, we favour a model based on interruption of histone recycling. Parental histone recycling forms an attractive basis for maintaining histone modifications through replication (Corpet & Almouzni, 2009) and would be sensitive to loss of processive replication. Several known features of histone management at the fork allow it to provide a basis for carrying transcriptional memory through replication. Parental H3/H4 tetramers are segregated equally between the two daughter strands during replication (Jackson & Chalkley, 1981) and are found reincorporated within a few hundred nucleotides of the fork itself (Sogo *et al*, 1986). It has also recently been demonstrated that evicted parental histones generally remain within about 400 bp of their original location when reincorporated (Radman-Livaja *et al*, 2011), crucial if the histone modifications are to be kept in register with the underlying DNA sequence. There must also be mechanisms by which the information on the parental tetramers is transferred to the naïve histones incorporated during replication. Such mechanisms have been proposed for modifications associated with transcriptional repression, such as H3K9me3 and H3K27me3 (Bannister *et al*, 2001; Hansen *et al*, 2008; Margueron *et al*, 2009), but the situation regarding histone modifications in active genes is less clear. A recruitment-copying mechanism, analogous to that for H3K9me3 and H3K27me3, has been proposed based on the ability of WDR5 to recognise methylated H3K4 while forming a complex with several H3K4 COMPASS methyltransferase complexes (Wysocka *et al*, 2005). Alternatively, segregation of parental H3K4me3 to the daughter strands may be initially sufficient to maintain active transcription, which would then reinforce the mark through RNA polymerase II recruiting a COMPASS complex (Krogan *et al*, 2003; Ng *et al*, 2003). Both mechanisms would be potentially sensitive to loss of processive replication.

Two closely related models have been proposed for how interruption of histone recycling by replication fork arrest could lead to epigenetic instability. Groth and colleagues showed that, following treatment with hydroxyurea, H3 with parental histone modifications was displaced from chromatin in consequence of uncoupling of the replicative helicase and polymerase and buffered by the histone chaperone ASF1 (Jasencakova *et al*, 2010). They proposed that reinstallation of these marked histones could promote ectopic heterochromatinisation. This model has been invoked to explain an increase in heterochromatin formation in cells lacking the G4 helicase FANCJ (Schwab *et al*, 2013). However, we do not believe that this model accounts for the epigenetic instability we observe in *rev1* cells since, as noted above, we do not observe any evidence of heterochromatin formation either globally or locally in the Bu-1a locus of REV1-deficient cells. Rather, the results we present here can be adequately accounted for by our original model in which uncoupling of the helicase and leading strand polymerase gives rise to tracts of chromatin that passively loose their parental marks. This model can also account for both deactivation of expressed loci, such as Bu-1a studied here, and derepression of heterochromatic loci such as ρ-globin that we have previously reported (Sarkies *et al*, 2010).

Although it has been cogently argued that transcriptional memory is unlikely to rely on histone modifications as the turnover of nucleosomes, and of their modifications, is too high (Deal *et al*, 2010; Henikoff & Shilatifard, 2011), the data we present here are nonetheless most readily explained by a histone-based mechanism for transmitting epigenetic information on the transcriptional state of this gene. While it seems unlikely that such reliance on histone-based epigenetic memory would suffice in all circumstances, we suggest that this mechanism may be employed by terminally differentiated cells that would normally be unlikely to pass through more than a few cell divisions before entering G0 or apoptosing. This mechanism could become problematic when such cells become transformed. By gaining limitless replicative potential, the risk of histone recycling becoming locally perturbed would be likely to increase, the resulting ‘epimutations’ then contributing to the more rapid evolution of the tumour.

## Materials and Methods

### DT40 cell culture and mutants

DT40 cells were cultured as previously described (Simpson & Sale, 2003).

### Constructs & gene targeting

To delete the +3.5 G4 motif sequence from *BU-1,* we created a targeting construct by amplifying a genomic region between exon 1 and exon 4 with primers BU1SalF and BU1NotR (for oligo sequences see Supplementary Table S1). The PCR product was cloned into pBluescript and the region between *Hind*III and *Sac*I (Fig 2A) replaced with a linker containing unique *Mlu*I and *Bam*HI sites. A selection cassette conferring puromycin resistance (Arakawa *et al*, 2001) was inserted into the *Bam*HI site (Fig 2A). Drug-resistant clones were screened for targeted integration by digestion of genomic DNA with *Afl*II and *Mlu*I, followed by Southern blotting with a probe 5′ of the targeting construct generated with the primers BU1STHNF and BU1STHNR (Fig 2C). The knock-in constructs were generated by ligating the double-stranded oligos, listed in Supplementary Table S1, into the *Mlu*I site. A *Pvu*I site was added in front of the G4 motif in order to facilitate the screening of ligation products. The orientation of the G4 motif was checked by direct sequencing. For the generation of the spacer constructs, portions of the second intron of *BU-1* were amplified using the forward primers BU1SP1-3F and BU1SPR. The +3.5 G4 motif is included in the reverse oligo. The products were cloned into the *Mlu*I site of the +3.5 G4 targeting vector. The second intron of Bu-1a was selected as spacer DNA because the removal of the +3.5 G4 motif completely abrogates Bu-1a loss, excluding the presence of other sequences with significant potential to block the replication fork between the TSS and the +3.5 G4 motif.

### Bu-1a/b staining and fluctuation analysis for Bu-1a loss

Cells from cultures whose confluency was between 0.4 × 10^6^ and 2 × 10^6^/ml were directly stained for 10 min at room temperature with anti-Bu-1a conjugated with phycoerythrin (Santa Cruz clone 5K98, cat. no. 70447) at a 1:10 dilution and anti-Bu-1b directly conjugated to FITC (Santa Cruz clone 5K94, cat. no. 70449). Cells were analysed by flow cytometry using an LSRII cytometer (Becton-Dickinson). To carry out fluctuation analysis, single cells staining positive for Bu-1a were sorted using a MOFLO (Dako-Cytomation) sorting cytometer and grown for 20 generations before staining and analysis using flow cytometry as above (Supplementary Fig S3).

### Chromatin immunoprecipitation & antibodies

Chromatin immunoprecipitation (ChIP) was performed as previously described (Nelson *et al*, 2006) with minor modifications. Briefly, cross-linking was performed with 1% (v/v) formaldehyde for 10 min at room temperature. The reaction was quenched by the addition of glycine to a final concentration of 125 mM and incubation for 10 min at room temperature. Nuclei were extracted, resuspended in SDS buffer and sonicated 30 times for 30 s with 30-s intervals with a Bioruptor water bath sonicator (Diagenode). Following sonication, samples were diluted with IP dilution buffer (Nelson *et al*, 2006) and incubated overnight with the following antibodies: Histone H3 (Cell Signalling Technologies; cat number 2650), H3K9/14ac (Millipore; cat number 17-615), H3K4me3 (Cell Signalling; cat number 9727). H3K9me3 (Abcam; cat number ab8898), H3K36me3 (Abcam; cat number ab 9050). Normal rabbit IgG (Millipore) was used as negative control. Following washing steps, chromatin was reverse-cross-linked for purification of DNA. PCR primers for ChIP qPCR are listed in Supplementary Table S1.

### CD spectroscopy

Circular dichroism experiments were conducted on a Chirascan spectropolarimeter using a quartz cuvette with an optical path length of 1 mm. Oligonucleotide solutions were prepared at a final concentration of 10 μM in 10 mM lithium cacodylate (pH 7.2) containing 1 mM of EDTA and 100 mM of either LiCl or KCl. The samples were annealed by being heated at 95°C for 10 min and slowly cooled to room temperature. Scans were performed over the range of 200–320 nm at 20°C. Each trace is the result of the average of three scans taken with a step size of 1 nm, a time per point of 1 s and a bandwidth of 1 nm. A blank sample containing only buffer was treated in the same manner and subtracted from the collected data. The data were finally zero-corrected at 320 nm.

### UV melting transition determination

UV melting curves were collected using a Varian Cary 400 Scan UV-visible spectrophotometer by following the absorbance at 295 nm. Oligonucleotides solutions were prepared at a final concentration of 4 μM in 10 mM lithium cacodylate (pH 7.2) containing 1 mM EDTA and 100 mM KCl. The samples were annealed by heating to 95°C for 10 min and then slowly cooled to room temperature. Each sample was transferred to a 1-cm path-length quartz cuvette, covered with a layer of mineral oil, placed in the spectrophotometer and equilibrated at 5°C for 10 min. Samples were then heated to 95°C and cooled to 5°C at a rate of 0.5°C/min, with data collection every 1°C. Melting temperature (T_m_) values were obtained from the minimum of the first derivative of the melting curve.

### Determination of replication timing within BU-1

Wild-type DT40 cells were pulse labelled for 1 h with 50 μM BrdU, sorted into four S-phase fractions from early to late S-phase named S1 to S4 for qPCR analyses and two fractions (Early-S and Late-S) for DNA microarrays studies. Nascent strands were isolated by BrdU-immunoprecipitation and quantified by qPCR. For DNA microarrays, in order to obtain sufficient DNA, immunoprecipitated nascent strands were amplified by whole-genome amplification (WGA, Sigma) and then hybridised on Agilent Chicken Genome CGH Microarrays 4X180K, custom microarray design with genome reference Gallus gallus V3 May 2006 (Hassan-Zadeh *et al*, 2012).
