# Supplementary material for: Determinants of G quadruplex-induced epigenetic instability in REV1-deficient cells
Source: EMBO J. 2014 Sep 4;33(21):2507–20. doi: 10.15252/embj.201488398 (PMC4282387; doi:10.15252/embj.201488398)
Supplement: Supplementary file 1 [file embj0033-2507-sd1.pdf]

THE
EMBO
JOURNAL

Manuscript EMBO-2014-88398

Determinants of G quadruplex-induced epigenetic instability in REV1-deficient cells

Davide Schiavone, Guillaume Guilbaud, Pierre Murat, Charikleia Papadopoulou, Peter Sarkies, Marie-Noëlle Prioleau, Shankar Balasubramanian, Julian E. Sale

Corresponding author: Julian E. Sale, MRC Laboratory of Molecular Biology

Review timeline:

Submission date:	05 March 2014
Editorial Decision:	03 April 2014
Additional correspondence	24 April 2014
Revision received:	23 July 2014
Accepted:	13 August 2014

Editor: Hartmut Vodermaier

Transaction Report:

(Note: With the exception of the correction of typographical or spelling errors that could be a source of ambiguity, letters and reports are not edited. The original formatting of letters and referee reports may not be reflected in this compilation.)

1st Editorial Decision 03 April 2014

Thank you again for submitting your manuscript studying the role of the Bu-1 locus G4 motif in epigenetic instability. We have now received the reports from three expert referees, copied below for your information. As you will see, all referees acknowledge the overall interest of the topic as well as the potential importance of your new observations. However, they all remain unconvinced that the presented data are sufficiently complete and decisive to justify several of the key conclusions of the study, a concern that unfortunately prevents us from considering the paper suitable for rapid acceptance, but also from making any strong commitments regarding possible publication of an eventual revised manuscript at the current stage.

Given that all referees nevertheless remain overall supportive and interested in this work, I would however still like to give you an opportunity to address the key issues and provide more direct evidence for currently speculative conclusions by way of a major revision of this study, for which we in this case would be happy to offer an extended time frame of up to six months. For such a revision to be successful, it would be essential to strengthen the criticized experimental issues (especially the concerns regarding ChIP data repeats, controls and statistical significance), but also to incorporate the major experimental suggestions of referees 2 and 3 to more comprehensively assess gene expression and transcription-related histone marks. However, given the possibility of indirect effects and alternative models as explanations (based on the literature as indicated by refs 2 and 3), we strongly feel that it would also be important to solidify some of the other speculative conclusions in your current model, in particular the occurrence of large post-replicative gaps and ideally the lack of parental histone recycling. On the other hand, while the well-taken G4 stability and REV1 role-related queries of referee 1 should definitely by taken into consideration and clarified, I feel that this may not necessarily require addition of further experimental data.

Since it is our policy to allow only a single round of major revision (during which publication of any competing manuscript will have no negative impact on our final assessment of your revised study!), I would in this case be very helpful to hear back from you within the coming weeks with a detailed response letter and suggestions on how you could envision responding to these concerns and requests, in order to be able to further discuss (by phone or email) and define the requirements for a successful revision of this work.

Thank you for the opportunity to consider this work for The EMBO Journal - I look forward to hearing from you and to eventually receiving your revision.

_____

REFEREE REPORTS:

Referee #1:

In this manuscript, Sale and co-workers extend their analysis on G4-induced epigenetic instability and concomitant gene expression, by modifying one specific G4 motif in the Bu-1 locus. They demonstrate that in REV1-deficient cells the epigenetic instability of Bu1 is dependent on the presence of the endogenous G4 motif, but only when it is oriented in the leading strand and within 4kb of the TSS . Bu-1 instability can also be induced by other non-endogenous G4 motifs, although, of importance, so does a sequence that has only very limited capacity to fold into a quadruplex structure. Other observations of interest are i) that active transcription cannot override the local epigenetic disturbance induced by the G4 motif within the body of the gene, and ii) a peculiar but interesting trans effect where in an heterozygous situation the expression-instability of one allele induces loss of expression of the other allel (which by itself is devoid of the instability-causing motif).

In my opinion, the experiments are well designed, the data is very convincing and conclusive, and the manuscript provides important new insight into the biological consequences of G4 motives and the underlying mechanisms of epigenetic alterations in replication-compromised cells. The paper is very well written and I support its publication provided that more discussion is being given to some observations that are not necessarily in agreement with the author's model. Because the study is very hypothesis driven, the data are being interpreted with the model as a reference, however, while I think their model could be plausible, there are a number of observations that are not necessarily completely supportive. In my opinion, these concerns/points require more attention in the discussion section.

*) First and foremost, the notion that the motif with very poor G4 forming ability (Tm being below that of culturing conditions) is a capable inducer of epigenetic instability in Bu1 is concerning. It makes a dent in the hypothesis that it is a G4 structure as opposed to a yet unrecognized sequence motif that causes the instability, as it requires quite some imagination to consider it reasonable to assume that such a motif, just by virtue of its thermodynamic properties, would block ongoing DNA replication. More weight so be given that this outcome goes against a "structure" model: at 37 degrees there is no structure. I also think that the reasoning on page 7 "consistent with it creating a replication fork" is circular.

*) Also feeding the concern that other sequence features cannot be ruled out as determinants in the observed phenotype is the notion that some motifs that have very high Tm do not or hardly induce instability. The authors explain this by suggesting that REV1 does not act upon these and may be more important to aid replication of G4 structures with longer single stranded loops. But why do the more stable G4s do not cause instability in REV1 proficient cells (and in REV1 mutant cells)? If one were to think about redundancy of other resolving enzymes (e.g. helicases), why could those not act on the G4 motifs that apparently require REV1 and cause the problem in REV1 deficient cells? This explanation requires a remarkable specificity type in division of labor.

*) What are the arguments to place REV1 downstream of G4 structure formation? The authors propose that REV1 is needed to replicate through pre-existing G4 structures but what is the evidence or reasoning in favor of this hypothesis as opposed to placing REV1 upstream: that replication

impairment because of the absence of REV1 allows for a more frequent formation of G4 structures, which may by themselves induce epigenetic change (not necessarily via blocking fork movement) or more severe problems. If the latter hypothesis cannot be excluded through data, then the mentioning of the latter is warranted.

*) On a smaller note but related to that, is the expression of the deltaG4 allele in wildtype cells indeed lower than that of the normal allele (Figure 2B lower panel, black verus red line)? Is that statistically different?; it appears to be substantial. The clonal analysis displayed in Fig E3 may not be able to pick it up, although also here a little increase (or decreased expression) is seen and perhaps only the number of clones analyzed do not make it statistically different. If so, it may argue that the presence of a G4 motif affects expression (in wild type cells), independent of REV1 status, and perhaps REV1 deficiency just results in more frequent folding and thus a bigger effect. It would be interesting to know, whether this is a phenotype in which the other G4 alleles behave similar as in the Bu-1a loss phenotype in REV1-/- cells. If these alleles have been generated only in a REV1 deficient background, I do not suggest making them anew. However, if the "full-blown" expression of bu-1a in wildtype cells is dependent on the presence of the G4 motif, it should be discussed.

Minor point

It should be mentioned in the abstract which system/cells are being used as (as far as I know) the epigenetic instability phenotype has thus far only been observed in DT40 cells.

The notion that REV1 deficient cells are "defective" in replicating G-quadruplex forming DNA is too general and strong. If that were to be the case, cells would likely die instead of showing epigenetic instability. I don't know of any data that warrants such a bold statement. "Compromised " may be preferable.

Referee #2:

Unraveling mechanisms responsible for maintenance of epigenetic information in replicating cells is of broad interest in the field. This work builds up on the earlier work of the lab that proposed that replication block induced by G quadruplex (G4) sequences could interfere with transmission of epigenetic information on chromatin due to formation of post-replicative gaps. In this work, the authors gain further insights into how G4 sequences can affect transmission of an active transcriptional state, taking advantage of genetic manipulation of DT40 cells in a reporter Bu-1 locus containing G4 sequences on the coding DNA strand.
They observed that G4 located downstream of TSS (+3.5 G4 motif) is sufficient to induce epigenetic loss of reporter expression in cells lacking the REV1 translesion polymerase. They propose this is linked to replication forks progressing through the locus from the 3' end with the coding DNA strand replicated as the leading strand. The authors provide solid evidence that G4 sequence topology and Tm are not good predictive features for functional interference with transcription. Moreover, placing G4 away from its original position prevented Bu-1 expression loss. On this basis the authors suggest that if a zone with perturbed histone recycling does not include the gene promoter, the transcription would be maintained in cell progeny.
This is an interesting study with a solid functional analysis of G4 structures. However, the model remains speculative and there is no direct evidence of 1) altered transcription, 2) directional replication of the locus, 3) generation of large post-replicational gaps, and 4) lack of parental histone recycling. Moreover, strong conclusions are drawn from the ChIP analysis of histone modifications even though the differences are moderate and no statistical analysis is included (see below). Nevertheless, the manuscript deals with a very important question that is difficult to address and presents a solid genetic analysis of a phenomenon where transmission of an active state is jeopardized. Therefore this work is relevant for EMBO Journal and could be considered for publication if a few key points are addressed experimentally and the authors present a more open view on the potential mechanisms involved in this interesting phenomenon.

Major concerns
1. My major concern regards the conclusion that maintenance of active gene expression relies on proper recycling of active histone marks. However, there is not much support for this idea in the literature so far.

The ChIP data in panel 6C are not convincing and apparently repeated only twice on the same biological sample (according to legend). The analysis should be repeated in several biological replicas (starting with independent chromatin preps) in order to test whether the differences between the cell lines at individual sites are statistically significant.

The authors should also test whether the decrease of active marks accompanied by increase of silent marks such as for example H3K9me2/3 or H3K27me3?

In relation to point #2, the authors could do a ChIP of RNA pol II ser2P, ser5P as well as ChIP for H3K36me3 that is linked to transcriptional elongation to directly evidence changes in transcription.

Also, authors should discuss the possibility of G4 affecting transcription.

2. The level of gene expression is assessed in manuscript solely based on FACS analysis of the Bu-1 protein on cell surface, and thus only indirectly. However, the transcript levels were not analyzed to address directly the relationship between histone marks levels, expression and location of G4 from TSS. That would be necessary since data in Fig.6 are then used for one of the major conclusions of the paper and the model in Fig.7. (see also point#1)

3. The fluctuation analysis is not sufficiently described. For example, in graphical representation of data, do the data points refer to average values of single-cell clones (Fig. 2E, 3A, 5A, 6A)? The numbers of analyzed clones should be stated.

4. The major advance presented in this paper compared to previous work is 1) functional analysis of different G4 sequences, 2) evidencing that the distance of G4 to TSS is critical for its effect on transcriptional memory.
However, the title and model focuses on the effect of G4 replicational interference on maintenance of histone marks. Since this is not taken much further in the current ms compared to their previous work, a different title should be considered. Moreover, the authors strongly promote their model (also proposed in two previous papers) that the loss of transcriptional memory is due to 1) directional replication of the locus, 2) generation of large post-replicational gaps, and 3) lack of parental histone recycling. Yet given that there are no evidence for this (the large post-replicative gaps were observed in yeast after UV), a more open discussion would be appropriate. In particular, it is surprising that the authors do not discuss several other papers that also describe effects of perturbed replication on histone marks and silencing (Dubarry et al., 2011; Jasencakova et al., 2010; Schwab et al., 2013; Zaratiegui et al., 2011).

Minor issues:

1. Do 63 observations in inter-origin distance measurements (Figure E1C) refer to number of analyzed origins or number of fibers?

2. Material and methods - determination of replication timing (p15) - pulse length and BrdU concentration are not stated.

References:
Dubarry, M., I. Loiodice, C.L. Chen, C. Thermes, and A. Taddei. 2011. Tight protein-DNA interactions favor gene silencing. Genes Dev. 25:1365-1370.
Jasencakova, Z., A.N. Scharf, K. Ask, A. Corpet, A. Imhof, G. Almouzni, and A. Groth. 2010. Replication stress interferes with histone recycling and predeposition marking of new histones. Mol Cell. 37:736-743.
Schwab, R.A., J. Nieminuszczy, K. Shin-ya, and W. Niedzwiedz. 2013. FANCJ couples replication past natural fork barriers with maintenance of chromatin structure. The Journal of cell biology. 201:33-48.
Zaratiegui, M., S.E. Castel, D.V. Irvine, A. Kloc, J. Ren, F. Li, E. de Castro, L. Marin, A.Y. Chang, D. Goto, W.Z. Cande, F. Antequera, B. Arcangioli, and R.A. Martienssen. 2011. RNAi promotes heterochromatic silencing through replication-coupled release of RNA Pol II. Nature. 479:135-138.

Referee #3:

© European Molecular Biology Organization

Comments for Schivavone et al.,

The authors studied a well characterized locus Bu-1, which displays epigenetic instability in REV1-deficient cells due to the presence of G4 motifs. They found that a single G4 motif 3.5 kb downstream of the TSS was sufficient to create the epigenetic instability of this locus. And they further reported that such epigenetic instability was independent of the thermal stability of the G4 motifs, but was dependent on the position of the G4 motif. Finally, the authors attempted to establish a role of the G4 motif in controlling the histone modification pattern at its nearby regions. Most of the results are quite convincing, except the last part regarding the G4 motif's role in controlling histone modifications. I suggest the authors to further strengthen this part and also rephrase certain overstatements regarding this point, before this manuscript can be accepted by EMBO J.

Major points:
1. The title is absolutely an overstatement. "Control of histone modification...", I honestly do not see that. The effect on histone modification is first of all solely based on a ChIP assay, which is often questioned due to its variations. Moreover, such effect could very well be indirect and there is no evidence whatsoever for a direct "control". I strongly suggest the authors to change their title to a much more solid statement.

2. I suspect that the functional G4 motif affected the chromatin assembly and/or compaction at nearby regions in cells experienced the transcriptional change. And histone modification changes observed in the manuscript may be subsequent indirect events. To clarify this, mapping the nucleosome occupancy and chromatin accessibility at the region of interest in cells display distinct transcriptional states will be of help.

Other points:
1. Fig. 5A. Do all these G4 motifs induce similar replication fork stalling?

2. Other transcription related modifications, especially H3K27me3 and H3K9me2 should be investigated, because such induced repression events are most frequently associated with these modifications.

3. The authors entertained a model that the maintenance of "active" modification is required for keeping genes active. This is a popular view. But more and more experts studying chromatin and transcription hold a very different opinion. I suggest the authors to read a review paper discussing such issue (Trends Genet. 2011; 27: 389-396).

1st Revision - authors' response 23 July 2014

Thank you again for obtaining three insightful reviews of our paper so promptly and for your helpful letter. We now return a thoroughly revised version of our manuscript in which we have addressed experimentally all the points that we are able to tackle. I have detailed the changes we have made and the data we have added in the accompanying 'response to the reviewers'.

Following the suggestion of two of the reviewers we have changed the title of the paper to 'Determinants of G quadruplex-induced epigenetic instability in REV1-deficient cells', which we believe does not overemphasise just one aspect of the paper.

Concerning the points you highlighted in your letter:

1. The ChIP data: The ChIP data we presented in our original submission was in fact derived from two biological replicates, with triplicate qPCR determinations from each (i.e. 6 technical replicates). We have now expanded this to three biological replicates (independent IPs) for each data point (i.e. 9 technical replicates). We also now show the positive and negative controls for each antibody (revised Figure 7) and the H3 density (Figure E4). The latter shows no significant variation dependent on the position of the G4 motif in the locus. We have included statistical tests of the ChIP

qPCR data [Yuan et al., 2006, BMC Bioinformatics 7, 85] and indicated points that are highly significantly different to wild type.

2. Further analysis of gene expression and transcription-related marks. We have added ChIP data for H3K9me3 and H3K36me3 for the G4 spacer experiments. There is no increase in H3K9me3, consistent with our previous observations (revised Figure 7C). Further, we see no evidence of increased chromatin compaction either globally or specifically in the Bu-1a locus in a newly added MNase assay (new Figure E5) suggesting that the loss of expression we observe is not due to heterochromatin formation. This is supported by the H3 density plot (new Figure E4). We tried to monitor Ser2 and Ser5 phosphorylation of RNAPII, but despite multiple attempts we were unable to obtaining convincing enrichment, even in control loci. However, we have obtained data for H3K36me3. Interestingly, this mark is not affected directly by the position of the G4 motif, like H3K4me3 and H3K9/14ac, but rather seems to simply track transcriptional activity, as previously observed.

In terms of the link between the transcription of Bu-1a and its expression, we have already shown that there is a direct link between the percentage of Bu-1a^{low} variants in a population and the mRNA levels as determined by qPCR [Sarkies et al 2012 NAR Figure 3E & F]. We have also added qPCR data for the spacer mutants, which confirms that transcript levels are the same as wild type despite the significant changes in gene body histone marks when the G4 motif is at +4.5 kb from the TSS.

Concerning the criticism that our model is speculative, our model is just that, a working hypothesis. It does invoke a number of as yet incompletely understood, and frequently contentious, concepts including the *in vivo* behaviour of G quadruplex structures, the nature of histone management at the replication fork and the link between histone post-translational modifications and the transcriptional state of a locus. Referee 2 points out that there are two aspects of our proposed model for which we do not provide direct evidence: the existence of long, post-replicative gaps and histone recycling at the *BU-1* locus. These are of course important features of our underlying hypothesis, but there are significant technical and theoretical reasons why a convincing direct demonstration of these phenomena at the level of a single locus is likely to be extremely difficult or impossible. We have tried a number of techniques to detect single stranded DNA directly in the Bu-1a locus of REV1-deficient cells, as discussed in detail in the response to the reviewers, but failed to detect any signal above background, as we predicted might be the case in my last email. As we set out in the paper, our Monte Carlo simulation of Bu-1a loss, based on replication-dependent stochastic generation of Bu-1a loss variants, predicts that the frequency with which gaps long enough to reach the promoter of Bu-1a in the order of 0.02 per division, i.e. they form in about 2% of S phases. We have, however, mentioned our attempts to detect ssDNA directly and extensively revised our discussion of this issue.

Similar limitations apply to attempts to detect histone recycling at the Bu-1a locus. We have considered a number of different techniques, but I am not aware that anyone has got these to work at a single locus rather than at the level of bulk populations of cells [e.g. Jasencakova et al. Mol Cell 2010] or, using pulse-labelling, in whole single cells [e.g. Ray-Gallet et al. Mol Cell 2011]. We therefore do not believe this is a point that can be readily tackled directly with current technology.

Referee #1:

In this manuscript, Sale and co-workers extend their analysis on G4-induced epigenetic instability and concomitant gene expression, by modifying one specific G4 motif in the Bu-1 locus. They demonstrate that in REV1-deficient cells the epigenetic instability of Bu1 is dependent on the presence of the endogenous G4 motif, but only when it is oriented in the leading strand and within 4kb of the TSS. Bu-1 instability can also be induced by other non-endogenous G4 motifs, although, of importance, so does a sequence that has only very limited capacity to fold into a quadruplex structure. Other observations of interest are i) that active transcription cannot override the local epigenetic disturbance induced by the G4 motif within the body of the gene, and ii) a peculiar but interesting trans effect where in an heterozygous situation the expression-instability of one allele induces loss of expression of the other allele (which by itself is devoid of the instability-causing motif).

In my opinion, the experiments are well designed, the data is very convincing and conclusive, and the manuscript provides important new insight into the biological consequences of G4 motives and the underlying mechanisms of epigenetic alterations in replication-compromised cells. The paper is very well written and I support its publication provided that more discussion is being given to some observations that are not necessarily in agreement with the author's model. Because the study is very hypothesis driven, the data are being interpreted with the model as a reference, however, while I think their model could be plausible, there are a number of observations that are not necessarily completely supportive. In my opinion, these concerns/points require more attention in the discussion section.

**) First and foremost, the notion that the motif with very poor G4 forming ability (Tm being below that of culturing conditions) is a capable inducer of epigenetic instability in Bu1 is concerning. It makes a dent in the hypothesis that it is a G4 structure as opposed to a yet unrecognized sequence motif that causes the instability, as it requires quite some imagination to consider it reasonable to assume that such a motif, just by virtue of its thermodynamic properties, would block ongoing DNA replication. More weight so be given that this outcome goes against a "structure" model: at 37 degrees there is no structure. I also think that the reasoning on page 7 "consistent with it creating a replication fork" is circular.*

This is an important point, but I think it can be argued both ways! We show for the endogenous +3.5 G4 motif that its *in vivo* effect is related to its ability to form an *in vitro* quadruplex structure. G4#1 also forms a clear *in vitro quadruplex*, but with a low melting temperature. However, we currently have no real idea how the biophysical behaviour of a short oligonucleotide in a test tube translates into what happens in the genomic context when other factors are present. While we cannot prove that a quadruplex is forming *in vivo*, we can say that the sequence is producing a biological effect very similar to other G-quadruplex forming sequences that we study. We now consider these ideas in more detail in the Discussion and add the caveat suggested by the reviewer.

**) Also feeding the concern that other sequence features cannot be ruled out as determinants in the observed phenotype is the notion that some motifs that have very high Tm do not or hardly induce instability. The authors explain this by suggesting that REV1 does not act upon these and may be more important to aid replication of G4 structures with longer single stranded loops. But why do the more stable G4s do not cause instability in REV1 proficient cells (and in REV1 mutant cells)? If one were to think about redundancy of other resolving enzymes (e.g. helicases), why could those not act on the G4 motifs that apparently require REV1 and cause the problem in REV1 deficient cells? This explanation requires a remarkable specificity type in division of labor.*

It is not the case that the more stable G4s cause no instability, just less. This can perhaps be seen more clearly in the original FACS data than in the scatter plots we presented. We now show this raw data as new supplementary new Figure E2. The Bu-1a^{low} populations can clearly be seen in cells with G4#4 (bottom centre), although their magnitude doesn't reach statistical significance and they are certainly not as striking as for G4 #1 (middle, centre), the +3.5 G4 (middle, left) or the rho-globin G4 (bottom, right). However, there is a clear difference when compared with the G4 knockout (top, right) and wild type (top, left). We do believe these results will turn out to be important in understanding the behaviour of G4s *in vivo.* We do have some evidence from work in other mutants that there is specificity in the division of labour in processing different G4 motifs, but at the moment this is preliminary and not within the scope of the present paper. We have modified our description of this point with reference to the raw data now shown in Figure E2.

**) What are the arguments to place REV1 downstream of G4 structure formation? The authors propose that REV1 is needed to replicate through pre-existing G4 structures but what is the evidence or reasoning in favor of this hypothesis as opposed to placing REV1 upstream: that replication impairment because of the absence of REV1 allows for a more frequent formation of G4 structures, which may by themselves induce epigenetic change (not necessarily via blocking fork movement) or more severe problems. If the latter hypothesis cannot be excluded through data, then the mentioning of the latter is warranted.*

This is an important point. We did in fact cover in an early draft of the paper and have now reinstated it as a Discussion point. Briefly, it unlikely that REV1-deficiency is upstream of the formation of G quadruplex structures i.e. facilitates their formation by creating more single stranded

DNA. REV1-deficient cells do not exhibit any global change in replication fork dynamics on undamaged DNA [Edmunds et al. 2008 Mol Cell; Jansen et al 2009, MCB] nor do they exhibit any spontaneous increase in single stranded DNA or checkpoint activation [Jansen et al 2009, MCB]. Thus, there is not a generalised impairment of replication in the absence of REV1 and therefore unlikely to be any significant additional opportunity for G4 formation.

**) On a smaller note but related to that, is the expression of the deltaG4 allele in wild type cells indeed lower than that of the normal allele (Figure 2B lower panel, black verus red line)? Is that statistically different?; it appears to be substantial. The clonal analysis displayed in Fig E3 may not be able to pick it up, although also here a little increase (or decreased expression) is seen and perhaps only the number of clones analyzed do not make it statistically different. If so, it may argue that the presence of a G4 motif affects expression (in wild type cells), independent of REV1 status, and perhaps REV1 deficiency just results in more frequent folding and thus a bigger effect. It would be interesting to know, whether this is a phenotype in which the other G4 alleles behave similar as in the Bu-1a loss phenotype in REV1-/- cells. If these alleles have been generated only in a REV1 deficient background, I do not suggest making them anew. However, if the "full-blown" expression of bu-1a in wildtype cells is dependent on the presence of the G4 motif, it should be discussed.*

While there is slight variation between clones of both wild type and *rev1* cells in their expression of Bu-1a, these differences are not significant as they are within the intrinsic inter-clonal variability we see with our antibody labelling protocol. The range of this variability, into which the example shown in Figure 2B clearly falls, can be seen in the new Figure E2, in which we present examples of raw data generated from different clones. This slight variability in the mean expression of the Bu-1a positive population is taken into account when gating for the Bu-1a loss variants, as explained in the legend to new Figure E2 and E3. Further, as the referee suggests, we have removed the +3.5 G4 from both alleles of wild type cells and this confirms that the G4 motif has no direct impact on expression of Bu-1a. We show this result in new Figure E6.

Minor point

It should be mentioned in the abstract which system/cells are being used as (as far as I know) the epigenetic instability phenotype has thus far only been observed in DT40 cells.

Yes, this is true (and something we are, of course, planning to address in future studies). The cell system in now mentioned in the abstract.

The notion that REV1 deficient cells are "defective" in replicating G-quadruplex forming DNA is too general and strong. If that were to be the case, cells would likely die instead of showing epigenetic instability. I don't know of any data that warrants such a bold statement. "Compromised " may be preferable.

OK, changed.

Referee #2:

Unraveling mechanisms responsible for maintenance of epigenetic information in replicating cells is of broad interest in the field. This work builds up on the earlier work of the lab that proposed that replication block induced by G quadruplex (G4) sequences could interfere with transmission of epigenetic information on chromatin due to formation of post-replicative gaps. In this work, the authors gain further insights into how G4 sequences can affect transmission of an active transcriptional state, taking advantage of genetic manipulation of DT40 cells in a reporter Bu-1 locus containing G4 sequences on the coding DNA strand.
They observed that G4 located downstream of TSS (+3.5 G4 motif) is sufficient to induce epigenetic loss of reporter expression in cells lacking the REV1 translesion polymerase. They propose this is linked to replication forks progressing through the locus from the 3' end with the coding DNA strand replicated as the leading strand. The authors provide solid evidence that G4 sequence topology and Tm are not good predictive features for functional interference with transcription.

Moreover, placing G4 away from its original position prevented Bu-1 expression loss. On this basis the authors suggest that if a zone with perturbed histone recycling does not include the gene promoter, the transcription would be maintained in cell progeny.
This is an interesting study with a solid functional analysis of G4 structures.

However, the model remains speculative

We agree completely – the model we advance remains speculative - but in our view it thus far best explains the data. We are studying a process that we cannot directly visualise or recapitulate *in vitro* and therefore we have to probe the system indirectly. Our model makes a number of assumptions and some of these assumptions are not amenable to direct testing, at least in any robust way. However, like all good models it makes a number of predictions, some of which we test in the current paper. The results do take our understanding of this phenomenon forward and lay the ground for further studies. We have, of course, considered other possible explanations for our observations and have now significantly revised the Discussion to consider these points.

With regard to the reviewer's views on what is not directly shown:

and there is no direct evidence of

1) altered transcription,

We have previously provided clear evidence that loss of Bu-1a expression as monitored by cytometry reflects a change in the level of Bu-1a transcript in REV1-deficient cells. The level of mRNA in a population correlates directly with the degree of loss of surface Bu-1a [Sarkies et al 2012 NAR Figure 3F, shown below].

F

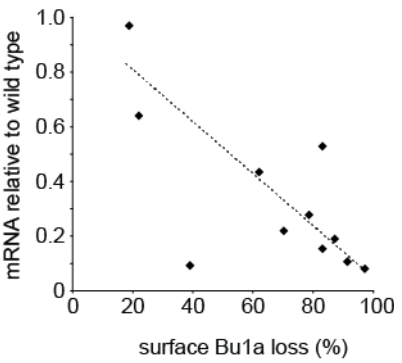

We will include a clearer reference to this data and also shown the relative mRNA levels for the cell lines used in the spacer experiments as new Figure E4B.

2) directional replication of the locus,

We provided a detailed characterisation of the replication of the locus in the original manuscript and show that it is likely that it is replicated from the 3' end in approximately 50% of S phases. The fact that deletion of the +3.5 G4 motif completely stabilises the expression of Bu-1a in REV1-deficient cells, and only induces epigenetic instability when on the coding rather than template strand (relative to the Bu-1a transcriptional unit) is consistent with epigenetic instability resulting only when replication enters from the 3' end of the locus. Thus, while the locus may be replicated from the 5' side, this does not account for the instability. Importantly the model does not require that a locus be *only* replicated from one direction. The model predicts that whether the locus is unidirectionally replicated from the 3' end or bidirectionally replicated would not affect whether Bu-1a loss occurs, but would affect its frequency. This is something that we are currently hoping to test using polar fork barriers, but is beyond the scope of this paper. Another point for further study is whether histone mark loss actually potentiated by the bi-directional replication of the locus. Thus, one could imagine that there would be a high likelihood that a post-replicative gap formed by a stall at the +3.5 G4 motif would be replicated by a fork coming from the 5' end of the locus. This idea would predict

that the gap may not persist for very long, but nonetheless be replicated in the absence of a supply of parental histones and is potentially relevant to the next point.

3) generation of large post-replicational gaps,

This existence of post-replicative single strand gaps is a prediction of our model, but one that is tricky to demonstrate directly. As we note in the paper, we believe that these gaps are actually relatively uncommon events in REV1-deficient cells. The Monte Carlo simulation of Bu-1a loss based on our model suggests that our observations are consistent with a frequency of gaps long enough to reach the promoter of Bu-1a in the order of 0.02, i.e. we predict that they form in about 2% of S phases. Thus, even in a synchronised population of cells, their prevalence will be low, even assuming that they persist for a reasonable time. As discussed above, this may not be the case given the bi-directional replication of the locus. Thus, we did not believe that any signal would rise significantly above background of currently available methods of direct detection. Nonetheless, we have considered and / or attempted a number of approaches to directly detect the putative single strand gaps in this locus.

1. Immunoprecipitation of single stranded DNA with RPA ChIP: There is no anti-chicken RPA antibody currently available. Despite identifying an anti-human antibody that worked well in denaturing Western blots, we could not get it to immunoprecipitate native chicken RPA.

2. Mapping ssDNA by random priming: This technique has been used to map ssDNA at stalled replication origins [Feng et al. (2007) Methods, 41, 151-157] and relies on the principle that the Klenow I fragment is able to amplify only from a single stranded template. We isolated DNA without denaturation and then incubated the DNA with Klenow fragment and biotinylated dCTP and then isolated the amplified regions with streptavidin-coated magnetic beads. This is quite a noisy technique and, using qPCR, we were unable to detect any significant increase in signal over background using a range of primers spanning -1 kb to +4 kb from the TSS.

3. We also tried to enrich ssDNA with an anti-ssDNA antibody [Millipore, cat no. MAB2024] or by immunoprecipitation of BrdU-containing DNA without denaturation [Azuara (2006, Nat. Protocols, 1, 2171-2177]. However, again we were unable to detect a significant increase in qPCR signal between *rev1* and wild type cells.

However, for the reasons given above, I really do not think that failure of these techniques refutes the model we propose, but reflects that we are probably not dealing with an abundant and / or long-lived ssDNA intermediate. We have discussed these points further in the revised paper and included the caveat that we have so far been unable to directly detect these structures.

4) and lack of parental histone recycling.

This is something that is, I believe, currently impossible to robustly demonstrate at the level of a single locus. The general concept of parental histone recycling is well established experimentally in a number of systems and has been extensively reviewed. We have previously argued that the relative preservation of H4 tail acetylation in the absence of H3K4me3 and H3K9/14ac is consistent with biased new histone incorporation at the normally repressed rho-globin locus. However, this argument cannot readily be used at an active locus, in which the H4 tail will be acetylated anyway. (The specific predeposition H4 acetylations at K5 and K12 are also seen in transcriptionally active loci). Indeed, we have previously shown that loss of Bu-1a is associated with loss of H4 tail tetra-acetylation (along with loss of H3K4me3 and H3K9/14ac) [Sarkies et al 2012 NAR], but it is difficult to interpret this result in terms of deactivation of the locus as opposed to new histone incorporation.

We have also considered various approaches to pulse labelling newly synthesised histones, which has worked well in whole cell-level or cell population-level experiments [e.g. Ray-Gallet et al Mol Cell 2011]. However, I am not aware of such a method working robustly at the level of a single locus. Indeed, it is quite unlikely that it would be possible to get a decent signal to noise ratio with such an experiment. Even if one could label all the newly synthesised histones in the space of a single cell cycle, the difference between a normally replicated locus and one with biased new

histone incorporation would be 50% vs 100%. However, we are only expecting that difference in a small number of cells and in practice only a fraction of the total new H3 in the cell will be labelled. Thus, it is very unlikely that it would be possible to detect a specific signal.

Moreover, strong conclusions are drawn from the ChIP analysis of histone modifications even though the differences are moderate and no statistical analysis is included (see below). Nevertheless, the manuscript deals with a very important question that is difficult to address and presents a solid genetic analysis of a phenomenon where transmission of an active state is jeopardized. Therefore this work is relevant for EMBO Journal and could be considered for publication if a few key points are addressed experimentally and the authors present a more open view on the potential mechanisms involved in this interesting phenomenon.

See below for discuss of the ChIP analysis.

Major concerns
1. My major concern regards the conclusion that maintenance of active gene expression relies on proper recycling of active histone marks. However, there is not much support for this idea in the literature so far.

We agree that there is currently more support for histone mark recycling playing a role in the maintenance of heterochromatic states. However, there is evidence to support a role for H3K4me3 in maintaining fully active transcription through replication (e.g. Muramoto et al Curr Biol 2010; Ng & Gurdon NCB 2008) and, as we discuss in the paper, plausible mechanisms by which recycling of the mark could promote its propagation to newly incorporated histones after replication. Our work provides the evidence that processive replication can be required to locally maintain the mark. If the mark was simply a installed as a passive consequence of transcription then we would not predict that interruption of processive replication would have any impact on its distribution. However, we observe that the pattern of H3K4me3 is affected primarily by the position of the replication block and not by the transcriptional activity of the gene, particularly in when the G4 motif is at the +4.5 kb position. This suggests strongly that processive replication is required to maintain H3K4me3, rather than the G4 having any direct effect on a histone modifying enzyme or transcription itself. Thus, the processes that could be directly affected are limited. The most logical target is histone management at the fork and we believe that this forms the basis for the simplest explanation for our data.

Importantly, we would not wish to claim that this is necessarily the only way in which active transcription is maintained. We suspect one of the problems is that the mechanisms that preserve transcriptional states are very context dependent and that there are multiple ways of achieving the same outcome. There is a relative paucity of data on this topic as the link between replication impediments and maintenance of epigenetic states has not been extensively studied: our previous papers and those cited by the reviewer below actually cover much of the literature on the subject. Of course, our suggestions to explain our data remains hypotheses and I hope that our broader discussion makes this clear.

The ChIP data in panel 6C are not convincing and apparently repeated only twice on the same biological sample (according to legend). The analysis should be repeated in several biological replicas (starting with independent chromatin preps) in order to test whether the differences between the cell lines at individual sites are statistically significant.

The ChIP data we provided in Figure 6C of the original manuscript was derived from two independent clones for each knock-in line and therefore are from biological replicates with independent chromatin preps. We have further strengthened the existing data by now presenting the mean of *three* biological replicates for each data point for each ChIP, which equate to nine technical replicates. We also now explicitly include the controls we performed, enrichment at control loci and the IgG background (new Figure 7), and the H3 density (Figure E4). We also indicate statistically significant data points, which include the loss of H3K4me3 and H3K9/14ac in the body of the gene in the +1.0 kb spacer mutant.

The authors should also test whether the decrease of active marks accompanied by increase of silent marks such as for example H3K9me2/3 or H3K27me3?

We have previously shown that loss of Bu-1a expression in *rev1* cells does not appear to result in a significant increase in H3K9me2 at the promoter of the locus [Sarkies et al 2012 NAR]. However, we appreciate that this is an important point, particularly given other models for replication-dependent epigenetic instability have been proposed that do suggest that H3K9me3 might be installed [e.g. Jasencakova et al 2010 Mol Cell; Schwab et al 2013 JCB]. We have now done H3K9me3 ChIP our set of G4 spacer mutants and the results confirm that for *rev1* cells there is no installation of this repression-associated mark (new Figure 7C), consistent with the observation of some residual expression of Bu-1a in the Bu-1a^{low} population [Sarkies et al 2012 NAR]. We have also added a global and Bu-1a-specific MNase assay that shows that there is no increase in compaction of the locus excluding heterochromatin formation in this context.

In relation to point #2, the authors could do a ChIP of RNA pol II ser2P, ser5P as well as ChIP for H3K36me3 that is linked to transcriptional elongation to directly evidence changes in transcription.

We have tried extensively to get phospho-RNAPII ChIPs to work and cannot obtain convincing enrichment with the antibodies we have used. However, we have examined H3K36me3 (new Figure 7D), which as expected shows an enrichment in the body of the gene. REV1-deficient cells exhibit a reduction in the mark in agreement with the reduced expression levels of Bu1a in this cell line. Cells with the G4 at +4.5 kb and at +6.0 kb display normal levels of H3K36me3. Thus, in contrast to H3K4me3, the level and pattern of H3K36me3 does appear to track expression rather than the position of the G4 motif. We have revised the Discussion to incorporate these observations.

Also, authors should discuss the possibility of G4 affecting transcription.

We did discuss the issue of this G4 affecting transcription directly, for instance by it forming part of an intragenic regulatory element, on page 10 of the original manuscript. Its presence has no effect on the overall level of expression of Bu-1a, which we now show in new Figure E6, but it does affect the rate with which expression is lost in REV1-deficient cells.

2. The level of gene expression is assessed in manuscript solely based on FACS analysis of the Bu-1 protein on cell surface, and thus only indirectly. However, the transcript levels were not analyzed to address directly the relationship between histone marks levels, expression and location of G4 from TSS. That would be necessary since data in Fig.6 are then used for one of the major conclusions of the paper and the model in Fig.7. (see also point#1)

As noted above, we have previously clearly established the link between surface Bu-1a expression and mRNA levels [Sarkies et al 2012 NAR]. However, we now present this data for the experiments shown in Figure 6.

3. The fluctuation analysis is not sufficiently described. For example, in graphical representation of data, do the data points refer to average values of single-cell clones (Fig. 2E, 3A, 5A, 6A)? The numbers of analyzed clones should be stated.

We have explained the fluctuation analysis more clearly and added a new supplementary figure (Figure E3). Each point on the scatter plots represents an individual clone expanded from a Bu-1a positive state, and shows the percentage of Bu-1a loss in that clone after a fixed time. We have added 'n' for the number of clones analysed to the graphs.

4. The major advance presented in this paper compared to previous work is 1) functional analysis of different G4 sequences, 2) evidencing that the distance of G4 to TSS is critical for its effect on transcriptional memory.
However, the title and model focuses on the effect of G4 replicational interference on maintenance of histone marks. Since this is not taken much further in the current ms compared to their previous work, a different title should be considered. Moreover, the authors strongly promote their model (also proposed in two previous papers) that the loss of transcriptional memory is due to 1) directional replication of the locus, 2) generation of large post-replicational gaps, and 3) lack of parental histone recycling. Yet given that there are no evidence for this (the large post-replicative gaps were observed in yeast after UV), a more open discussion would be appropriate. In particular, it is surprising that the authors do not discuss several other papers that also describe effects of

perturbed replication on histone marks and silencing (Dubarry et al., 2011; Jasencakova et al., 2010; Schwab et al., 2013; Zaratiegui et al., 2011).

We fully appreciate the referee's comments here and on reflection in the light of these comments and those of reviewer 3, we have given the paper a new and more general title that does not focus on just one aspect of the work.

Our work was meant primarily to test our working model using robust genetics and clear readouts because, as discussed above, direct demonstration of some of the key assumptions of the model is going to be extremely difficult. The original model has actually stood up well to the tests of it presented in the current work. However, we have extensively rewritten the Discussion to give a more open view in the context of other related work, as suggested, and co.

Minor issues:

1. Do 63 observations in inter-origin distance measurements (Figure E1C) refer to number of analyzed origins or number of fibers?

This is the number of inter-origin distances computed, from 67 analysed fibres. We have now mentioned this in Figure E1.

2. Material and methods - determination of replication timing (p15) - pulse length and BrdU concentration are not stated.

We have added add this information to Figure E1, but the protocol was fully described in Hassan-Zadeh et al. PLoS Biol 2012.

References:
Dubarry, M., I. Loiodice, C.L. Chen, C. Thermes, and A. Taddei. 2011. Tight protein-DNA interactions favor gene silencing. Genes Dev. 25:1365-1370.
Jasencakova, Z., A.N. Scharf, K. Ask, A. Corpet, A. Imhof, G. Almouzni, and A. Groth. 2010. Replication stress interferes with histone recycling and predeposition marking of new histones. Mol Cell. 37:736-743.
Schwab, R.A., J. Nieminuszczy, K. Shin-ya, and W. Niedzwiedz. 2013. FANCJ couples replication past natural fork barriers with maintenance of chromatin structure. The Journal of cell biology. 201:33-48.
Zaratiegui, M., S.E. Castel, D.V. Irvine, A. Kloc, J. Ren, F. Li, E. de Castro, L. Marin, A.Y. Chang, D. Goto, W.Z. Cande, F. Antequera, B. Arcangioli, and R.A. Martienssen. 2011. RNAi promotes heterochromatic silencing through replication-coupled release of RNA Pol II. Nature. 479:135-138.

Referee #3:

Comments for Schivavone et al.,

The authors studied a well characterized locus Bu-1, which displays epigenetic instability in REV1-deficient cells due to the presence of G4 motifs. They found that a single G4 motif 3.5 kb downstream of the TSS was sufficient to create the epigenetic instability of this locus. And they further reported that such epigenetic instability was independent of the thermal stability of the G4 motifs, but was dependent on the position of the G4 motif. Finally, the authors attempted to establish a role of the G4 motif in controlling the histone modification pattern at its nearby regions. Most of the results are quite convincing, except the last part regarding the G4 motif's role in controlling histone modifications. I suggest the authors to further strengthen this part and also rephrase certain overstatements regarding this point, before this manuscript can be accepted by EMBO J.

Major points:
1. The title is absolutely an overstatement. "Control of histone modification...", I honestly do not see that. The effect on histone modification is first of all solely based on a ChIP assay, which is often

questioned due to its variations. Moreover, such effect could very well be indirect and there is no evidence whatsoever for a direct "control". I strongly suggest the authors to change their title to a much more solid statement.

As discussed above in the response to reviewer 2, we have revised the title so as not to concentrate on just one aspect of the work.

2. I suspect that the functional G4 motif affected the chromatin assembly and/or compaction at nearby regions in cells experienced the transcriptional change. And histone modification changes observed in the manuscript may be subsequent indirect events. To clarify this, mapping the nucleosome occupancy and chromatin accessibility at the region of interest in cells display distinct transcriptional states will be of help.

We did assess nucleosome occupancy by measuring total H3 around the TSS. Our ChIP experiments were normalised to this, but we did not show the data explicitly and we now do this in new Figure E4. The position of the G4 motif has no readily discernable impact on the overall nucleosome density around the TSS. We have also carried out global and locus-specific MNase mapping (new Figure E5) and observe no difference between wild type and *rev1* cells, showing that loss of REV1 does not result in increased heterochromatinisation.

Other points:
1. Fig. 5A. Do all these G4 motifs induce similar replication fork stalling?

We are really not sure how this could be measured in a meaningful way *in vivo*. Ideally we would use 2D electrophoresis, but this is not currently feasible at a single copy locus in DT40.

2. Other transcription related modifications, especially H3K27me3 and H3K9me2 should be investigated, because such induced repression events are most frequently associated with these modifications.

We have added new data to address this point (new Figure 7), as discussed in the response to reviewer 2.

3. The authors entertained a model that the maintenance of "active" modification is required for keeping genes active. This is a popular view. But more and more experts studying chromatin and transcription hold a very different opinion. I suggest the authors to read a review paper discussing such issue (Trends Genet. 2011; 27: 389-396).

We are very aware of this paper, which provides a considered and careful review of the topic and of the divergent opinions concerning the role of histone marks in maintaining active gene expression. We have no particular wish to jump into either camp, but it seems to us that the most straightforward explanation for our data is that at least one histone modification is contributing to the specification of the expression state of this locus and that impeded replication is somehow interfering with this. We have extensively rewritten the discussion to hopefully provide a broader perspective to our results.

Editorial Decision 13 August 2014

Thank you for submitting your revised manuscript for our consideration. It has now been seen once more by two of the original referees (see comments below), and I am happy to inform you that they are happy with the revisions and have no further objections towards publication in The EMBO Journal!

Thank you again for this contribution to The EMBO Journal and congratulations on a successful publication! Please consider us again in the future for your most exciting work.

Referee #1

In the revised version of the manuscript, the authors addressed all the issues I raised adequately/satisfactorily. In addition, they went to great length addressing the concerns raised by the other reviewers, either, when possible, experimentally, or through further explanation/elaboration, or by nuancing their discussion.

It is indeed true that for some of the assumptions onto which the model is build there is not (yet) a lot of experimental support, but I think that is not at all an obstacle that would preclude publication. In general, in cases where frequencies of biological events are low, many of the current technologies will simply not be applicable.

I think it is an excellent study, with great genetics, aiming to address an important biological problem as to how cells maintain their histone code when having to deal with naturally occurring replication impediments.

Referee #3

I have no further issue and I recommend to publish this paper.

© European Molecular Biology Organization
