# Supplementary figures and images for: Determinants of G quadruplex-induced epigenetic instability in REV1-deficient cells
Source: EMBO J. 2014 Sep 4;33(21):2507–20. doi: 10.15252/embj.201488398 (PMC4282387; doi:10.15252/embj.201488398)

**A**

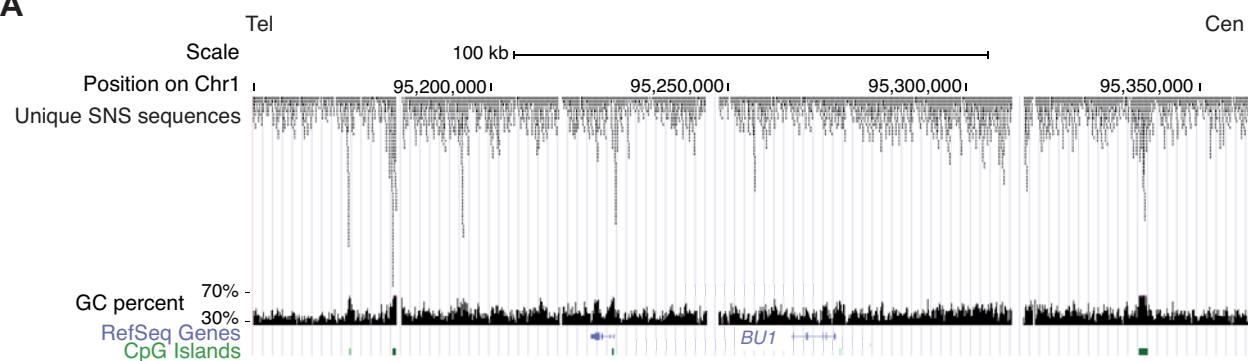

**B**

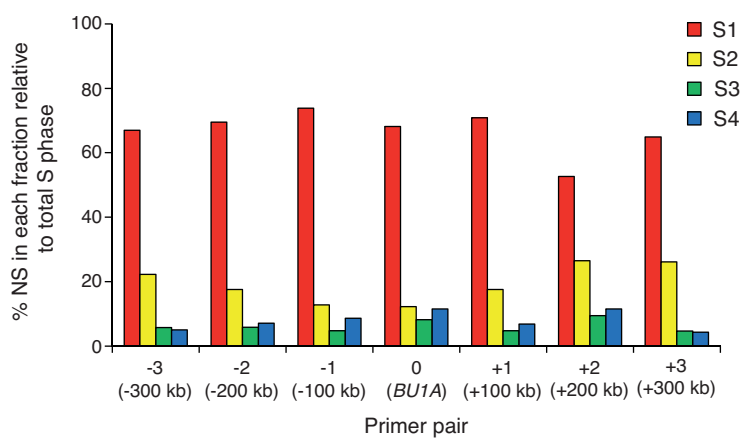

**C**

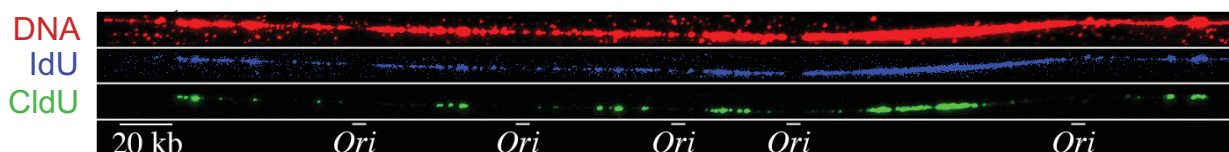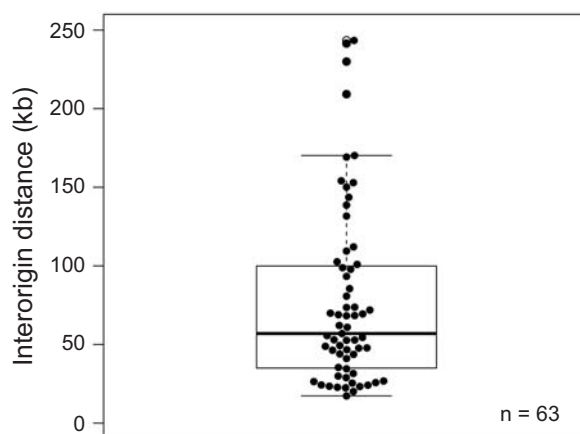

Supplement: Supplementary file 2 [file embj0033-2507-sd2.pdf]

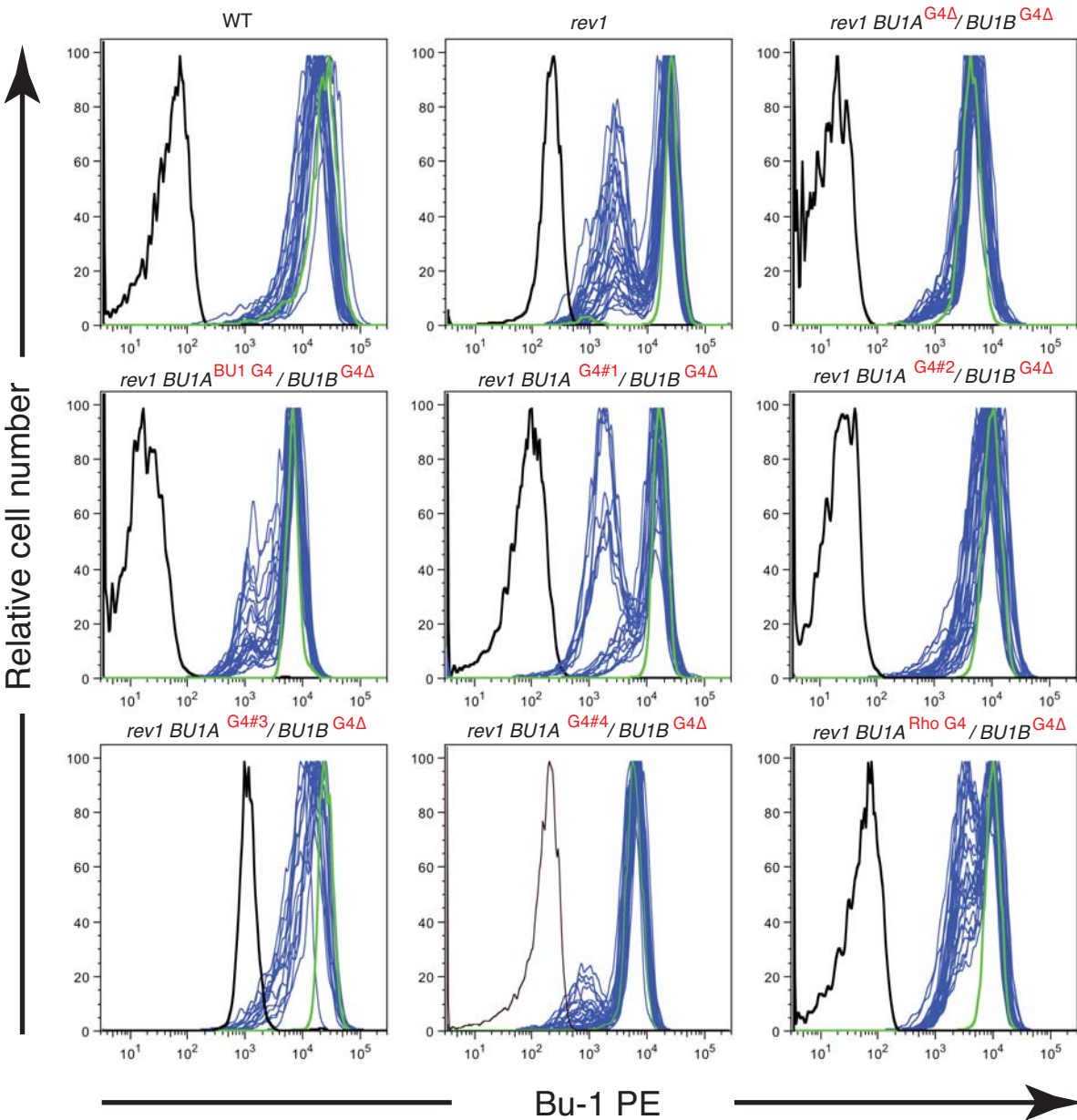

Supplement: Supplementary file 3 [file embj0033-2507-sd3.pdf]

A

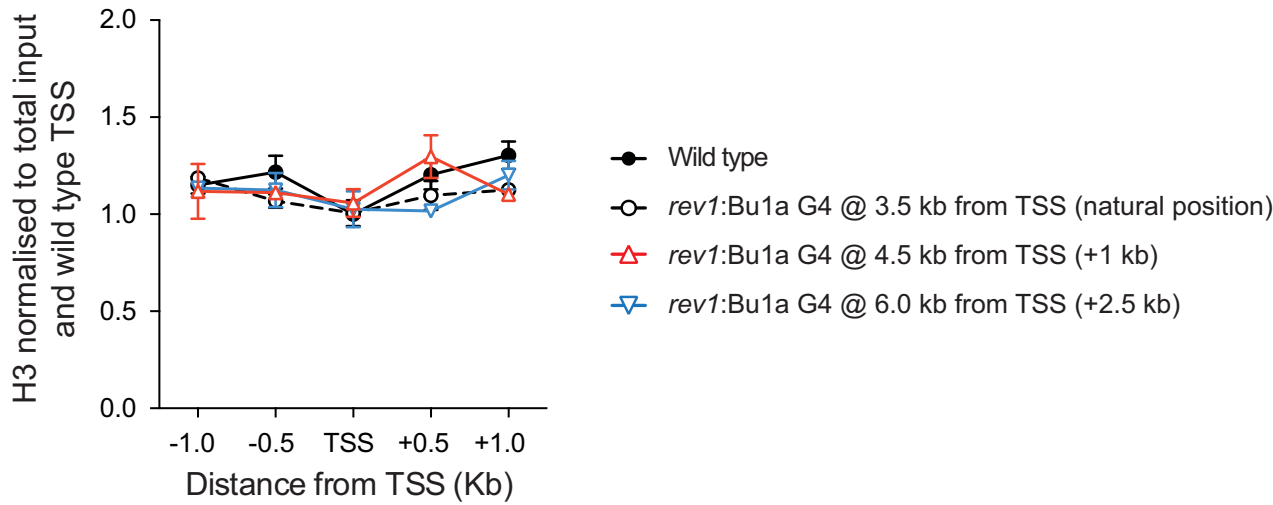

B

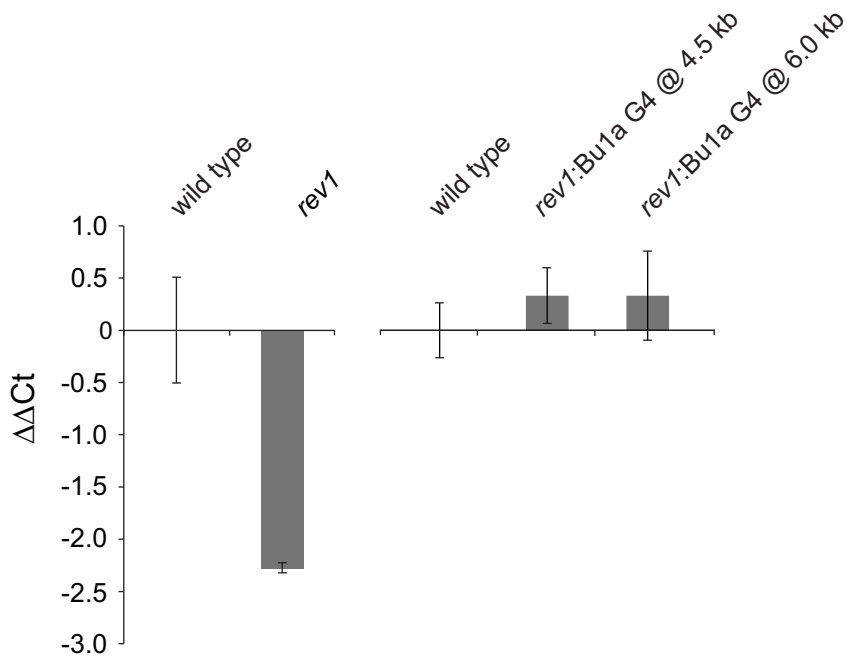

Supplement: Supplementary file 5 [file embj0033-2507-sd5.pdf]

A

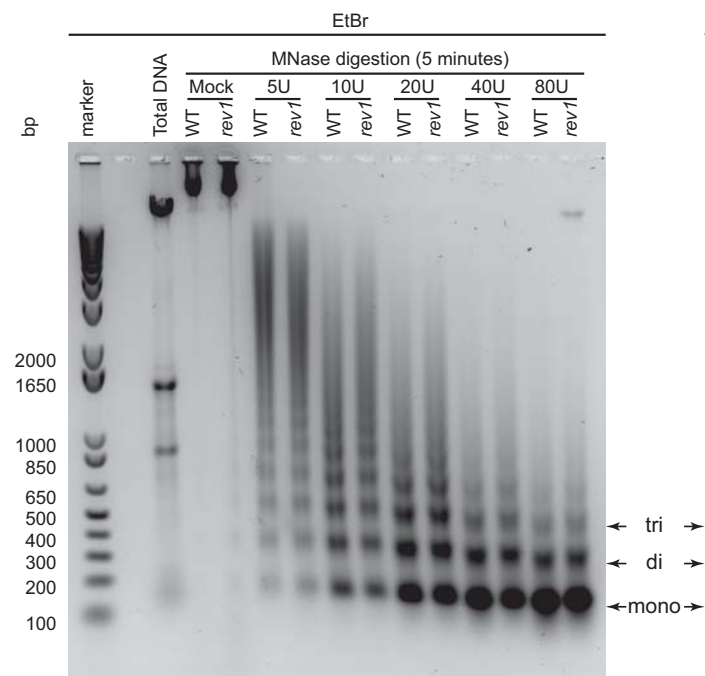

C

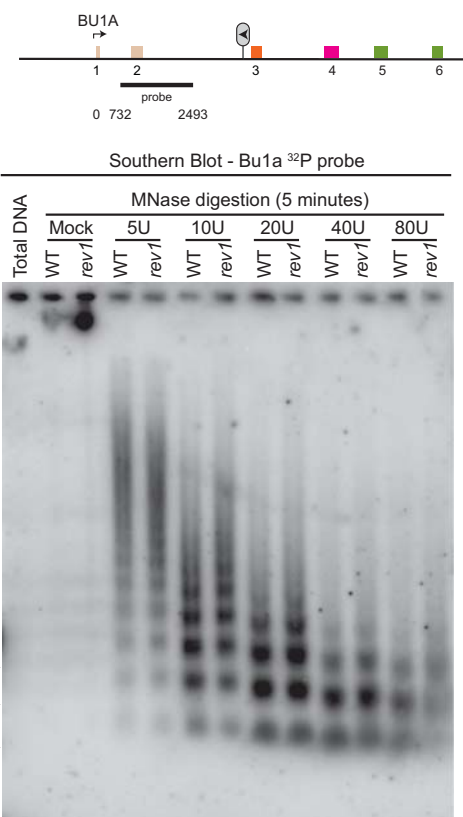

B

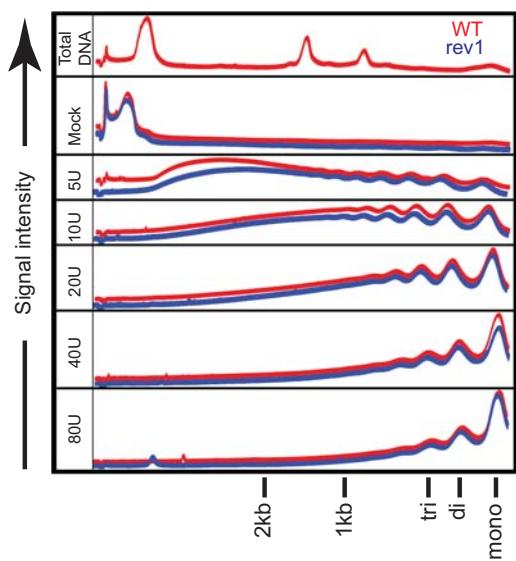

D

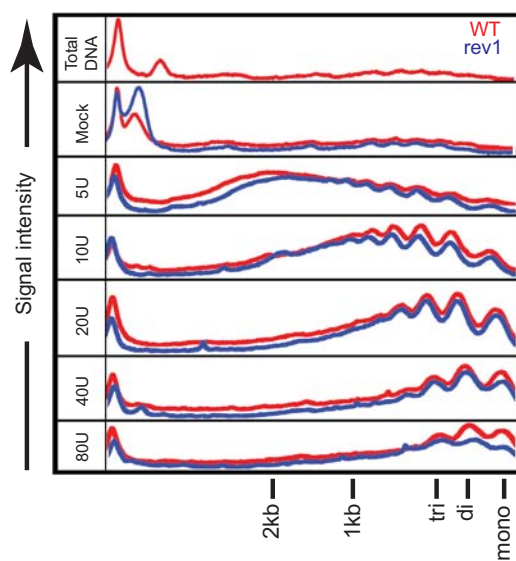

Supplement: Supplementary file 6 [file embj0033-2507-sd6.pdf]

**A**

wild type

BU-1 $\Delta$ G4

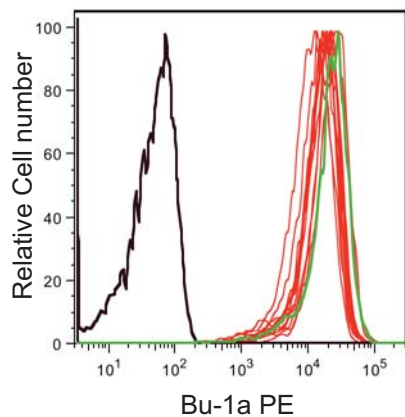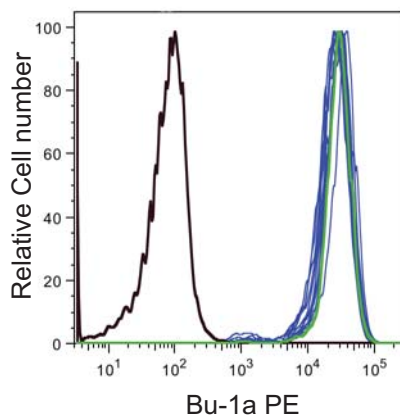

— wild type unstained  
— wild type stained

**B**

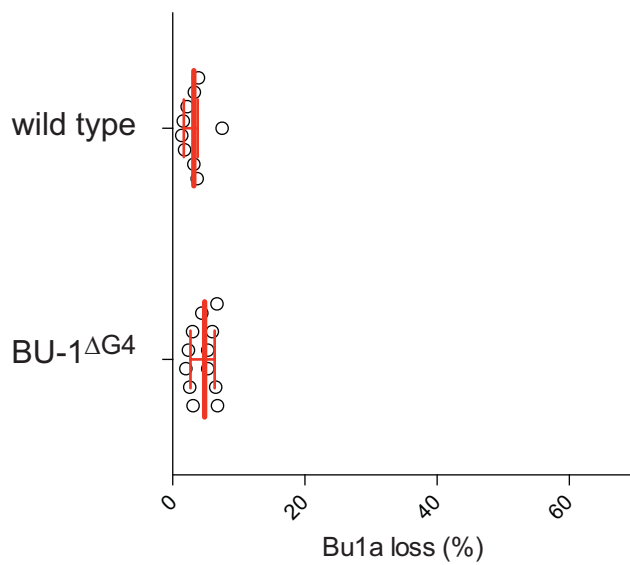

Supplement: Supplementary file 7 [file embj0033-2507-sd7.pdf]

**A**

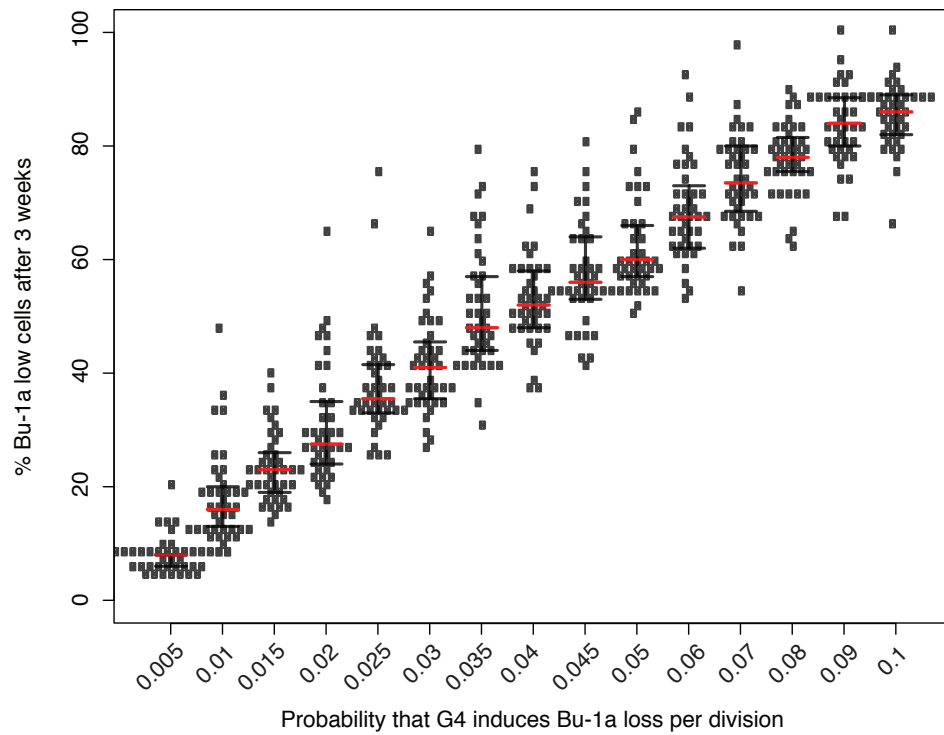

**B**

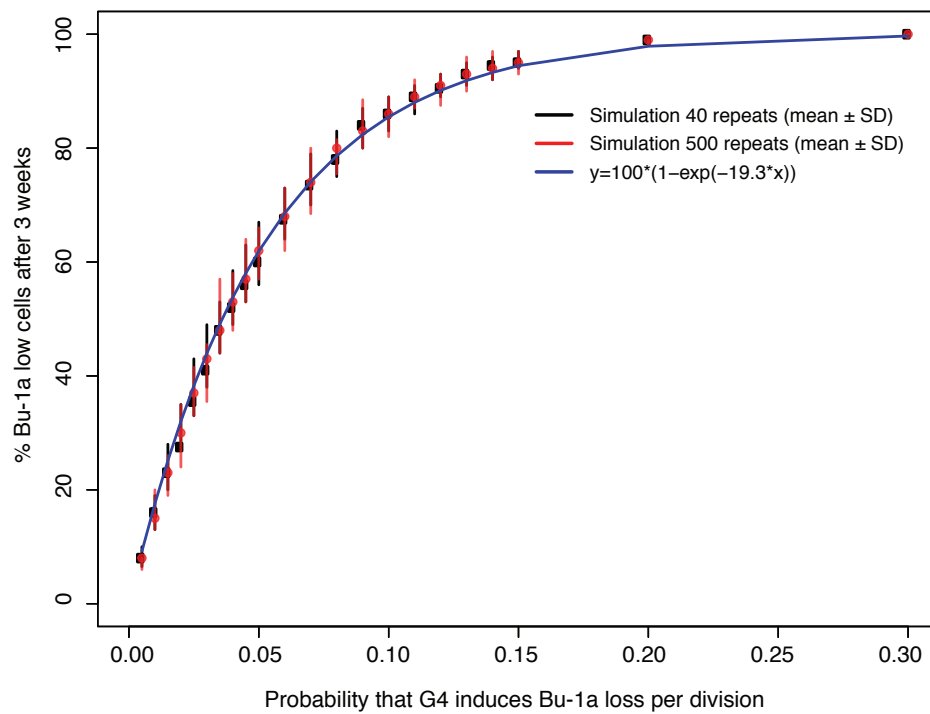

Supplement: Supplementary file 8 [file embj0033-2507-sd8.pdf]

## G4 @ TSS +6.0 kb

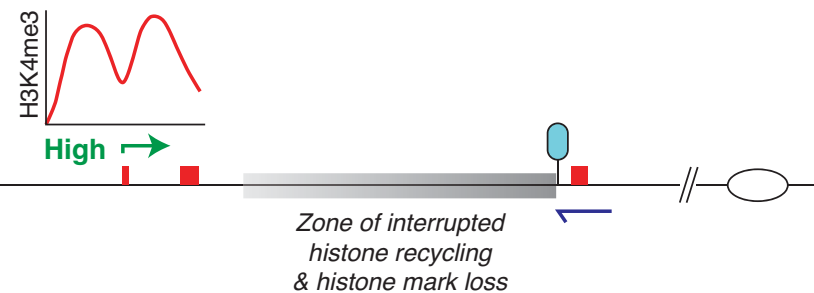

## G4 @ TSS +4.5 kb

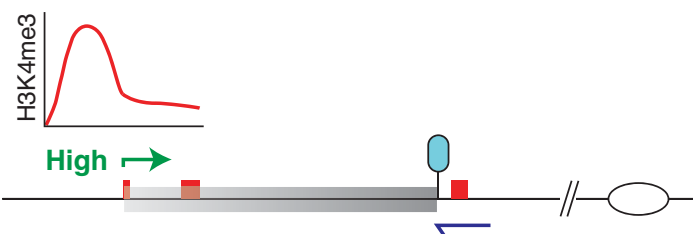

## G4 @ TSS +3.5 kb

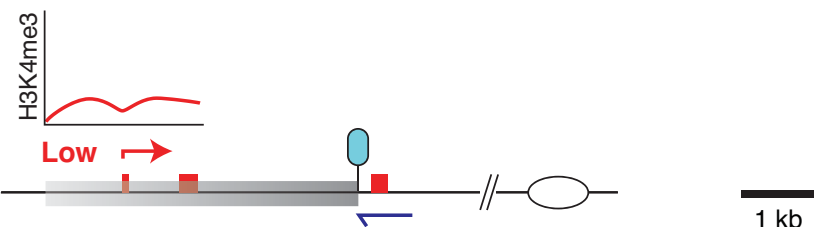

Supplement: Supplementary file 10 [file embj0033-2507-sd10.pdf]
