# Supplementary material for: Determinants of G quadruplex-induced epigenetic instability in REV1-deficient cells
Source: EMBO J. 2014 Sep 4;33(21):2507–20. doi: 10.15252/embj.201488398 (PMC4282387; doi:10.15252/embj.201488398)

Schiavone et al. Figure S3

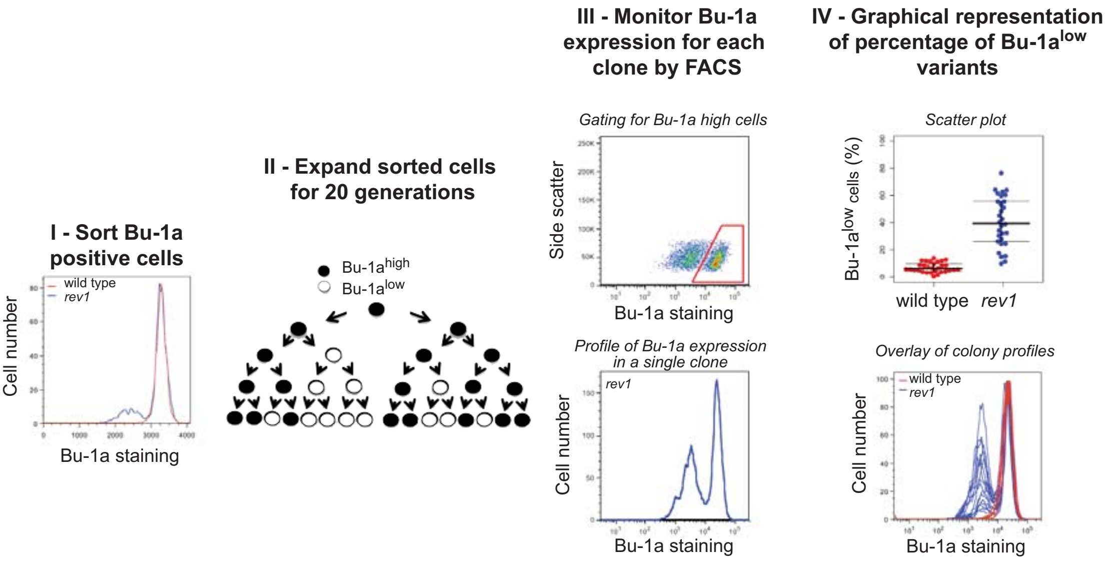

Supplement: Supplementary file 4 [file embj0033-2507-sd4.pdf]
