# Supplementary material for: Determinants of G quadruplex-induced epigenetic instability in REV1-deficient cells
Source: EMBO J. 2014 Sep 4;33(21):2507–20. doi: 10.15252/embj.201488398 (PMC4282387; doi:10.15252/embj.201488398)
Supplement: Supplementary file 9 [file embj0033-2507-sd9.pdf]

Figure S7C. R script for the Monte Carlo simulation of Bu-1a loss

```
# Developed by G.Guilbaud, Julian E Sale Lab.
# Simulation of the generation of Bu-1a loss variants in populations
 of Bu-1a positive DT40 cells

# Settings: (i) Number of repeats (clones) to be computed (ii) Number
 of starting Bu1a high cells (iii) Number of generations (iv)
 Probabilities tested (v) Optional Graphical output

## (i) Number of repeats (clones) to be computed

Number.of.repeat <- 500


## (ii) Number of cells Bu1a high to start with

starting.cells <- 2 # Number of starting Bu1a high cells

 # We sort 2 Bu1a positive cells per wells. As all cells do not grow
 in a well, the number of cell is not always of 2.
 # A 2 cells per well, we usually end up with half of the wells empty
 # In order to mimic experimental observation; we apply a probability
 that a cell will grow:

prob.cell <- 0.2 ### We experimentally computed that the probability
 that is to be use in Binomial distribution is 0.2 to match with
 experimental observation.


## (iii) Number of generations

Mean.number.of.division <- 19 # The number of division correspond of
 the number of cycle each cell undergoes. Typically for rev1 cells
 over 20 days = 19

SD.number.of.division <- 2  # In each well, we do not observe the
 same number of divisions. We observed that this number is normally
 distributed around a mean with as standard deviation of 2


## (iv) Probabilities tested:

prob.loss <- seq(0.00,0.2,0.005) #probability of loss tested


## (v) Optional Graphical output

graphics <- TRUE # If TRUE performs graphics
beeswarm <- TRUE # One the graphic require to install the beeswarm
 library, if want to avoid it set as FALSE
```

```
## Determine the number of generation for each repeat and number of
 cell per well

# Determine the number of generations for each well. Normally
 distributed (Mean=Mean.number.of.division, sd=SD.number.of.division)
N <- NULL
for (i in 1:Number.of.repeat){
 N.temp <-
 round(rnorm(1,Mean.number.of.division,SD.number.of.division),0)
 N <- c(N,N.temp)
}


## Determine the number of cell(s) per well

cell <- NULL

# As empty wells do not give results, we run the computation an
 arbitrary 10 times and select the first 'Number.of.repeat' non null
 observations to run the simulation

  for (i in 1:(Number.of.repeat*10)){

starting.cells.temp <- rbinom(1, starting.cells, prob.cell)

if(starting.cells.temp!=0){ cell <- c(cell, starting.cells.temp)}

     }


## Select only length of repeat number required:

Cells <- cell[1:Number.of.repeat]


## Output table of number of generations and number of cells for this
 simulation

write.table(N,"Number of generations applied for this
 simulation.txt",sep="\t")
write.table(Cells,"Number of cell(s) per well for this
 simulation.txt",sep="\t")


## Set vector and table used for simulation
TabOut.detailed <- NULL
Tab.Out<- NULL


## Start simulation

for(p in prob.loss){  ## Loop to cover all probability tested
```

```
## Set vector inside p loop
All.percent <- NULL
Cells.BU1A.high <- rep(NA,times=Number.of.repeat)

for ( g in 1:Number.of.repeat) {  ## For a given probability repeat
 as many time as Number.of.repeat


## Set vector inside g loop
TabOut.temp.2 <- NULL

Cells.BU1A.high.first.round <- Cells[g] # Take number of Bu1a high
 cell to start as computed above
Cells.BU1A.high.this.round <- Cells.BU1A.high.first.round # Initiate
 Number of cell to start with above value, then will increase as (2^i
 - negative) cells at each cycle


for (i in 1:N[g]){ ##Compute evolution of loss for a given repeat, as
 many time as number of cycle


 ## Compute cell division
 Cells.number <- 2*Cells.BU1A.high.this.round


 ## Apply random generation of Bu1a low cell according to the
 probability tested
     State.of.BU1A.expression <- rbinom(Cells.number,1,p)   # 0
 corresponds to starting point = High Bu1a, 1 = low Bu1a
     Cells.BU1A.high.this.round <-
 length(State.of.BU1A.expression[which(State.of.BU1A.expression==0)])
 #So if increase prob=> increase 'loss' bu1a


##Collect probability at each cycle
freq.temp <- 1-
 (Cells.BU1A.high.this.round/(Cells.BU1A.high.first.round*2^i))
 #Number of Bu1a low cell = Number of Bu1a High - total number of
 cells
TabOut.temp.1 <- c(freq.temp,i,p,g)
TabOut.temp.2 <- rbind(TabOut.temp.2, TabOut.temp.1)

     }


##Collect results at the end of one repeat
TabOut.detailed <- rbind(TabOut.detailed, TabOut.temp.2)
Tab.Out <- rbind(Tab.Out, TabOut.temp.1)

 }
```

```
}


##Output table
 # Named columns
colnames(TabOut.detailed) <- c ("Frequency of Bu1a
 low","Generation","Probability","repeat")
colnames(Tab.Out) <- c("Frequency of Bu1a low","Number of
 Generations","Probability","repeat")


## Write tables
write.table(TabOut.detailed,"Bu1a low cell frequency over each cell
 cycle for all probabilities tested (Detailed
 table).txt",sep="\t",row.names = F)
write.table(Tab.Out,"Bu1a low cell frequency results for all
 probabilities tested .txt",sep="\t",row.names = F)


## (v) Optional Graphical output

if (graphics==TRUE){


 ## Set up table to plot for each probability the median and Standard
 deviation
 graph <- NULL
 for (a in unique(Tab.Out[,3])){
 prob.loss <- prob.loss +1

 num <- which(Tab.Out[,3]==a)

 graph.temp <-
 cbind(median(Tab.Out[num,1])*100,sd(Tab.Out[num,1])*100, a)
 graph <- rbind(graph, graph.temp)

}

 pdf(paste("Plot median and SD of Bu1a low clones frequency per
 probability.pdf"),height=10,width= (length(prob.loss)*0.7)) #Output
 as pdf whose width vary depending of the number of probabilities
 tested

plot(graph[,3], graph[,1],type="l",ylab="% of Bu1a low
 Cells",xlab="Probability of instability per cell cycle",lwd=3,xaxt =
 "n",ylim=c(0,100))
 axis(1,unique(Tab.Out[, 3]))
segments(graph[,3], graph[,1]-graph[,2], graph[,3], graph[,1]+
 graph[,2],lwd=3)

mtext("Plot median and SD of Bu1a low clones frequency per
 probability",outer=T,cex=1.5)
```

```
dev.off()

if(beeswarm==TRUE){

    library(beeswarm)


##Set up table for beeswarm
beeswarm.graph <- NULL

Prob <- 0 # beeswarm requires to have one column corresponding at one
number for one condition, here conditions are the probabilities
tested

for (b in unique(Tab.Out[,3])){
 Prob <- Prob+1

 num <- which(Tab.Out[,3]==b)
 beeswarm.graph.temp <-
 cbind(round(100*Tab.Out[num,1],0),rep(Prob,length(Tab.Out[num,1])))
 beeswarm.graph <- rbind(beeswarm.graph, beeswarm.graph.temp)

}

 pdf(paste("Boxplot of Bu1a low clones
 frequency.pdf"),height=10,width= (length(prob.loss)*0.7))   #Output
 as pdf whose width vary depending of the number of probabilities
 tested

    beeswarm(beeswarm.graph[,1] ~ beeswarm.graph[,2],main=("Bu1a low
 clones frequency distribution for a given probability"), data =
 beeswarm.graph,  method = 'hex',col =4, pch=16, ylab = '% of Bu1a
 low cells',xlab="Probability of instability per cell
 cycle",ylim=c(0,100),labels=unique(Tab.Out[, 3]))

    boxplot(beeswarm.graph[,1] ~ beeswarm.graph[,2], data =
 beeswarm.graph, add = T,names = FALSE)

dev.off()


 }

}
```
