# Supplementary material for: Determinants of G quadruplex-induced epigenetic instability in REV1-deficient cells
Source: EMBO J. 2014 Sep 4;33(21):2507–20. doi: 10.15252/embj.201488398 (PMC4282387; doi:10.15252/embj.201488398)
Supplement: Supplementary file 11 [file embj0033-2507-sd11.pdf]

Table S1

Oligonucleotides

Oligos for genomic manipulations

Oligo Name	Sequence (5' to 3')
BU1SalF	AGCGTCGACCGGTCGACGTGCAGCTAGACCAGAGTAGGTATT
BU1NotR	AAAAATTTTTAAAAGCGGCCGCGGATCGATGGATCTCCATAGACAGATGAGGAC
BU1STHNF	TGCAACTGGGACAGTAGTACAGAG
BU1STHNR	ACTGTCACTTACCAAGTACCTCACC
G4#1F	CGCGTCGATCGTTGGTTTTGGTTTTGGTTTTGGTA
G4#1R	CGCGTACCAAAACCAAAACCAAAACCAACGATCGA
G4#2F	CGCGTCGATCGTGGGTTTGGGTTTGGGTTTGGGTA
G4#2R	CGCGTACCCAAACCCAAACCCAAACCCACGATCGA
G4#3F	CGCGTCGATCGTGGGGTTGGGGTTGGGGTTGGGGA
G4#3R	CGCGTCCCCAACCCCAACCCCAACCCCACGATCGA
G4#4F	CGCGTCGATCGTTTTGGGTGGGTGGGTGGGTTTTA
G4#4R	CGCGTAAAACCCACCCACCCACCCAAAACGATCGA
BU1G4F	CGCGTCGATCGGGGCTGGGTGGGTGCTGTCAAGGGCTGGGA
BU1G4R	CGCGTCCCAGCCCTTGACAGCACCCACCCAGCCCCGATCGA
RGLOBG4F	CGCGTCGATCGGGGGAGTAAAAGGGAGCGGGGTGCTGGGGA
RGLOBG4R	CGCGTCCCCAGCACCCCGCTCCCTTTTACTCCCCCGATCGA
BU1G4MUTF	CGCGTCGATCGGGGCTG**A**GTGGGTGCTGTCAAG**A**GCTGGGA
BU1G4MUTR	CGCGTCCCAGC**T**CTTGACAGCACCCAC**T**CAGCCCCGATCGA
BU1SP1F	ATAACGCGTCGATCGCCTCCAGTGACGGGGCATCCACAACC
BU1SP2F	ATAACGCGTCGATCGGGTCCTAATCTCCACTGTTCCTTTTACATAC
BU1SP3F	ATAACGCGTCGATCGGTAAGTGGCCCTGTTAGCTACAAGTAGC
BU1SPR	CAACTGGTTCACAGTGGTCATCCCGACCCACCCACGACAGTTCCCGACCCTGCGCATAT

ChIP qPCR primers

Oligo Name	Sequence (5' to 3')
Bu1a_-1kb_F	CAGAACCAGCACAACAGCAT
Bu1a_-1kb_R	CACAGCATGAGAGCCAAAGA
Bu1a_-0.5kb_F	AAGAGCCTTGCGAAGTTCAG
Bu1a_-0.5kb_R	TTTTCTGCAGTGCCAATGAC
Bu1a_TSS_F	CTCTGTAGCCAGATCGTCTTCTC
Bu1a_TSS_R	GTGTCAGCTCATCTAGGCAAATC
Bu1a_+0.5kb_F	CTGCGACAGCTTTCCTTTGT
Bu1a_+0.5kb_R	ACAGCAGGTCTTTTCCCAAA
Bu1a_+1kb_F	AATGTCCCCAAAATGAGCTG
Bu1a_+1kb_R	CCTCTTTTTCCACCCTCCTC
b-globin 8.99F	GGGCCCAATGAACCAGAAA
b-globin 8.99R	TGTTCCCCAGCAACGCA

b-globin 13.19F	GAATGTGTCCATCTGCCCTCAT
b-globin 13.19R	GGGAAGCCATCCCTGCA
b-globin 21.37F	CTCTGTGCTCAGCATCCTTCAAT
b-globin 21.37R	CCTTTCGGCACTTTCTTCCTTT
GAPDHpromF	TTGCCGAGCAAACAGAGG
GAPDHpromR	CCCCATCTTGAGGTTACGAC
GUSB_(upstream)_F	TGCGAATGGACTGGAACTCT
GUSB_(upstream)_R	CAGGGCAATTTTGGGGTGAA
GUSB_(2nd exon)_F	CGGATGGGTGTGGTATGAGA
GUSB_(2nd exon)_R	CGATGGAGTAGTAGTGGGCA
RPLP0_(upstream)_F	CAATGGGCTGCGCGAGAG
RPLP0_(upstream)_R	GAGGGCTCCGGGAAAGAAG
RPLP0_(2nd exon)_F	TGTTTCGTGGTGGGAGCG
RPLP0_(2nd exon)_R	GCATCATCGTGTTCTTCCCC

Primers for verification *BU-1* replication timing.
(2006 *Gallus gallus* genome build)

**Oligo Name**	**Sequence (5' to 3')**
- 100kb F (chr1 : 95161924-95161944)	CAGCATCCAAAAAGGAAGACA
- 100kb R (chr1 : 95162025-95162045)	GCTGGACGATTTCAGGTCAGT
- 200kb F (chr1: 95052581-95052600)	CGCCACTGACTGGAATTTGT
- 200kb R (chr1: 95052694-95052713)	ACATCCCCTTCTTGCCATCT
- 300kb F (chr1: 94966178-94966197)	CAACCCAAACCAAAGCTCAC
- 300kb R (chr1: 94966308-94966327)	CCTGGGAACTTGGGAAAGAA
+ 100kb F (chr1: 95381680-95381699)	GTGCCTGTCCTGGGGTATTT
+ 100kb R (chr1:95381767-95381786)	TCCCAACTCCAATGCTTGAC
+ 200kb F (chr1: 95474442-95474461)	TAGGGGGCAGGACTGAAACT
+ 200kb R (chr1: 95474564-95474583)	AAGAAAAGCAGGCAGCACAA
+ 300kb F (chr1: 95550739-95550758)	GCTGGCTGCCCTCTCTAAGT
+ 300kb R (chr1: 95550842-95550861)	GCAATGCACAAAGAGCAACA
BU1A F (chr1: 95262822-95262844)	CTCTGTAGCCAGATCGTCTTCTC
BU1A R (chr1: 95262969-95262991)	GTGTCAGCTCATCTAGGCAAATC
