# Supplementary material for: Determinants of G quadruplex-induced epigenetic instability in REV1-deficient cells
Source: EMBO J. 2014 Sep 4;33(21):2507–20. doi: 10.15252/embj.201488398 (PMC4282387; doi:10.15252/embj.201488398)
Supplement: Supplementary file 12 [file embj0033-2507-sd12.pdf]

Supplementary Legends

Figure S1. Replication of the *BU-1* locus. A. Replication origins mapping around the *BU-1* locus. Unique short nascent strand sequences that align around the *BU-1* locus on chromosome 1 reveal several potential origins of replication at around 50 kb either side of the locus, but none within it. Also indicated is the percentage GC content calculated in a rolling 5-base pair window, the location of RefSeq annotated genes and CpG islands. B. Precise quantification of replication timing profiles around the *BU-1* locus. The position 0 corresponds to the *BU-1* locus. Wild type DT40 were pulsed for 1 hour with 50 μM BrdU and sorted into four temporally distinct S phase compartments, between early and late (S1, S2, S3 and S4), by FACS. After performing a BrdU-IP, enrichment of replicated DNA in a given region is tested in each of the four fractions. qPCRs around *BU-1* show a strong enrichment in the S1 fraction, confirming that the locus is replicated early in S phase. The locations of the primers used are indicated relative to the *BU-1* locus and are also shown in Figure 1C. C. Inter replication origin distance in wild type DT40 determined by DNA combing. WT DT40 were pulsed sequentially with IdU (15 minutes) and CldU (15 minutes). After DNA combing, DNA was stained in red, IdU in blue and CldU in green with fluorescent antibodies (Guilbaud *et al*, 2011). Above, an example DNA fibre in which the position of the replication origins is mapped (*Ori*). Below, a box and whisker plot of the distances between consecutive origins, the thick line corresponds to the median and the bottom and top of the box are the first and third quartiles. Among the 63 observations (from 67 fibres analysed) we observed a mean value of 77 ± 7 kb (1 SD).

<u>Methods for Figure S1</u>

Isolation of Short Nascent Strands (SNS) and Deep Sequencing analysis

Short nascent strand (SNS) purification and deep sequencing of SNS was performed as described previously (Hassan-Zadeh *et al*, 2012).

DNA combing

Asynchronously growing wild type DT40 were labeled for 15 min with 25 μM IdU alone then labeled for another 15 min with 25 μM CIdU.
At the end of the labeling period (30 minutes), cells were put in ice cold PBS (1 volume of cells for 2 volumes of 1X PBS) and centrifuged at 250 g for 5 min at 4 ºC,

washed in ice-cold PBS, centrifuged again and fixed in 80% ethanol in 1X PBS. Cells were then counted and around 400,000 cells were taken and embedded in 0.5 % low-melting point agarose. Cell digestion, combing on silanised coverslips, staining and analysis were performed as previously described (Labit *et al*, 2008; Guilbaud *et al*, 2011), with minor modifications: DNA was denatured for 30 minutes in 2.5 N HCl, and CldU was detected using rat anti BrdU (AbD Serotec) at 1 in 750.

Figure S2. Examples of raw cytometry data for fluctuation analysis for generation of Bu-1a^{low} variants. As the data for each mutant is collected on different days, and often with different batches of antibody, a Bu-1a positive (green) and unstained (black) control are included in each analysis. The removal of the original G4 motif, or its replacement with different G4 motifs has no impact on the expression of the Bu-1a^{high} population relative to wild type cells. Note the slight clone-to-clone variation in the mean signal of the Bu-1a^{high} population is corrected for when analysing the proportion of Bu-1a^{low} variants (Figure S3).

Figure S3. Principle of the fluctuation analysis for the generation of Bu-1a^{low} variants. The approach is based on the classical analysis of Luria and Delbrück for monitoring the acquisition of phage resistance in *E. coli* (Luria & Delbrück, 1943). It allows quantification and comparison of the rate of a stochastic process. I. Bu-1a^{high} cells are sorted as single cells in 96 well plates and allowed to expand for a set time. II. During expansion, the culture stochastically generates Bu-1a^{low} variants. We have previously shown that once a cell has become Bu-1a^{low}, it remains stably low (Sarkies *et al*, 2012). III. The proportion of Bu-1a^{low} variants is assessed by cytometry by setting a gate that is centred on the mean of the Bu-1a^{high} population. The mean staining intensity of this Bu-1a^{high} population varies very slightly from clone to clone (Figure S3) and this gating approach corrects for this, as described previously for the analysis of surface immunoglobulin loss-variants in hypermutating B cell lines (Sale, 2012). IV. By analysing multiple clones in parallel it is possible to determine the median number of Bu-1a^{low} variants. The original Luria-Delbrück equations do not work well for the high rate of variant generation in this system and we have adopted a Monte-Carlo simulation approach to estimate a per-division probability of a cell transitioning from being a Bu-1a^{high} to Bu-1a^{low} state.

Figure S4. H3 density around the *BU-1* TSS and mRNA levels in wild type and in *rev1* mutants harbouring the +3.5 G4 in natural position 3.5 kb from the TSS, and at 4.5 kb and 6.0 kb. A. The H3 signal was normalised to total input. Error bars = 1SD of three independent immunoprecipitations. B. Bu-1 mRNA quantification in for the 4.5 kb and 6.0 kb spacer mutants. The *rev1* cells (with the natural +3.5 G4) used in the ChIP experiments were analysed previously (Sarkies *et al*, 2012) and the data are shown here for comparison. Error bars = SD for 3 technical replicates.

Figure S5. MNase assay for nucleosome density in wild type and *rev1* cells. The left hand panel shows DNA from wild type and *rev1* cells digested with the indicated number of units of MNase for 5 minutes and stained with ethidium bromide. The right hand panel is a Southern blot of the left hand gel probed for the Bu-1a locus as indicated in the map above the blot. The pattern of digestion, which indicates the spacing of nucleosomes, is quantified by densitometry and plotted below, indicate that there is no change in nucleosome density either globally (A & B) or locally (C & D).

Methods for Figure S5

MNase digestion and blotting

2x 10^8 cells were harvest and washed once in cold 1X PBS. Cell lysis and MNase digestion were performed as described (Carey & Smale, 2007), without restriction digestion, using the indicated units of micrococcal nuclease (Thermo Scientific) per 200 µL reaction. 5 µg of DNA were loaded for each condition in a 1% agarose gel and run at 2 V/cm in 1X TBE. Ethidium bromide staining was revealed by Fluorimager and the ^{32}P-dCTP signal by Phosphorimager (both using a Typhoon FLA 7000). Total DNA was obtained by overnight cell lysis at 37 ºC in 0.25 mg/mL proteinase K (Roche), 50 mM Tris-HCl, 5 mM EDTA, 0.5 % SDS. DNA was extracted with phenol-chloroform followed by a chloroform only step and precipitated with 10 % 3M NaAc pH5 and 1 volume of isopropanol. Southern blotting was performed using part of the 5' arm of the *BU-1* targeting construct (Figure 2A) as a probe (Figure S5C).

Figure S6. Deletion of the +3.5 G4 motif in wild type cells has no effect on the stability of expression of *BU-1*. Fluctuation analysis of clones of wild type DT40 cells or cells in which the +3.5 G4 motif has been removed from both alleles of the

BU-1 locus after expansion for 3 weeks. A. Overlaid cytometry plots showing that the +3.5 G4 motif does not affect the expression level or stability of Bu-1a. B. Quantification of Bu-1a^{low} variants.

Figure S7. Monte Carlo simulation of Bu-1a loss with the +3.5 G4 present in the *BU-1A* allele only. A. Output of simulation for forty clones with the results presented as a scatter plot mimicking the actual fluctuation analysis data presented in the main figures. Red bars = median; whiskers = interquartile range. B. Output of simulation for 500 clones (red bars) with a fitted non-linear regression curve of $y = 100(1\text{-exp}^{(-19.3x)})$. The data for the 40-clone simulation is shown for comparison (black bars). Points = median; whiskers = interquartile range. The equation can be used to estimate p (x axis) for a given observed median percentage of Bu-1a^{low} cells. C. R script for the Monte Carlo simulation of Bu-1a loss.

Methods for Figure S7

Computational modelling of Bu-1a loss

Calculation of a per division rate of mutation (or in this case epigenetically-induced loss of expression) is usually performed using the Luria-Delbrück equations, or derivatives thereof (Luria & Delbrück, 1943) However, these equations only work at rates of loss variant generation considerably lower than we observe for Bu-1. In order to estimate a per division probability (p) of a cell becoming Bu-1a^{low} we write a script in R (see below) to simulate Bu-1a loss as a function of p. In this model we apply a probability to each cell division that Bu1a expression will be reduced. According to experimental observations, all progeny of a Bu-1a^{low} cell will remain in this state (Sarkies *et al*, 2012). For a given probability we repeat the simulation n times, n representing the number of starting cells (in the examples shown n = 40 or n = 500). As in a real experiment, each 'clone' does not undergo the same number of divisions within a given time. We estimated the number of divisions as normally distributed with a standard deviation of 2, whose mean is computed as the number of hours of the experiment divided by the doubling time of the cells (an average of 22h for *rev1* cells in our experimental conditions in which the clone expands in a 96-well plate). Since the cloning efficiency of *rev1* cells is low (Simpson & Sale, 2003), we place 2 cells in each well. Poisson statistics suggest that, under these conditions, the majority of wells, but not all, in which cells grow have started from a single cell. This distribution is

incorporated into the script. The principal long-term difference between the model and experimental observation is that the model tends to 100% Bu-1a^{low} over time, which for *rev1* cells is rarely observed. This is likely explained by a slight competitive growth disadvantage for cells lacking high level Bu-1a expression, an effect may lead to a slight underestimation of the per-division rate of Bu-1a loss by the model.

Figure S8. Summary of the effect of G4 position on promoter histone modifications and transcription. A replication fork coming from the right (dark blue half arrow) is stalled at a G4 (blue lollipop) leading to formation of a post-replicative gap and a zone in which recycling of histones displaced by the replicative helicase is disrupted. The maximum length of this zone is determined by the maximum length of post-replicative gaps formed following the replication fork stall (and therefore on the maximum extent of dissociation of the helicase and polymerase). The data is consistent with this being fixed at ≤ c. 4 kb. Thus, as the G4 motif is moved closer to the TSS, the zone of histone mark loss first affects the body of the gene (middle panel), which does not affect transcription and then the promoter region, which does (lower panel). This suggests that the maintenance of histone modifications within *BU-1* is locally sensitive to the interruption of processive replication and therefore that the histone modifications are likely not only to facilitate transcription, but that in this context they also specify an active transcriptional state.

Table S1. Oligonucleotides.

Supplementary References

Carey M & Smale ST (2007) Micrococcal Nuclease-Southern Blot Assay: I. MNase and Restriction Digestions. *CSH Protoc* **2007:** pdb.prot4890

Guilbaud G, Rappailles A, Baker A, Chen C-L, Arneodo A, Goldar A, d'Aubenton-Carafa Y, Thermes C, Audit B & Hyrien O (2011) Evidence for sequential and increasing activation of replication origins along replication timing gradients in the human genome. *PLoS Comput. Biol.* **7:** e1002322

Hassan-Zadeh V, Chilaka S, Cadoret J-C, Ma MK-W, Boggetto N, West AG & Prioleau M-N (2012) USF binding sequences from the HS4 insulator element impose early replication timing on a vertebrate replicator. *Plos Biol* **10:** e1001277

Labit H, Goldar A, Guilbaud G, Douarche C, Hyrien O & Marheineke K (2008) A simple and optimized method of producing silanized surfaces for FISH and

replication mapping on combed DNA fibers. *BioTechniques* **45:** 649–52– 654–656–8

Luria SE & Delbrück M (1943) Mutations of Bacteria from Virus Sensitivity to Virus Resistance. *Genetics* **28:** 491–511

Sale JE (2012) Measurement of diversification in the immunoglobulin light chain gene of DT40 cells. *Methods Mol. Biol.* **920:** 417–432

Sarkies P, Murat P, Phillips LG, Patel KJ, Balasubramanian S & Sale JE (2012) FANCJ coordinates two pathways that maintain epigenetic stability at G-quadruplex DNA. *Nucleic Acids Res.* **40:** 1485–1498

Simpson LJ & Sale JE (2003) Rev1 is essential for DNA damage tolerance and non-templated immunoglobulin gene mutation in a vertebrate cell line. *EMBO J.* **22:** 1654–1664
